# Supplementary material for: Separating parental and treatment contributions to perinatal health after fresh and frozen embryo transfer in assisted reproduction: A cohort study with within-sibship analysis
Source: PLoS Med. 2021 Jun 25;18(6):e1003683. doi: 10.1371/journal.pmed.1003683 (PMC8274923; doi:10.1371/journal.pmed.1003683)
Supplement: S1 STROBE Checklist — (DOCX) [file pmed.1003683.s001.docx]

STROBE Statement—Checklist of items that should be included in reports of ***cohort studies***

|  | Item No | Recommendation | Page number | Relevant text from manuscript |
| --- | --- | --- | --- | --- |
| **Title and abstract** | 1 | (*a*) Indicate the study’s design with a commonly used term in the title or the abstract |  | Title, abstract (methods and findings) and first section of methods |
|  |  | (*b*) Provide in the abstract an informative and balanced summary of what was done and what was found |  | Abstract: methods and findings |
| Introduction | | |  |  |
| Background/rationale | 2 | Explain the scientific background and rationale for the investigation being reported |  | Introduction, first and second paragraph |
| Objectives | 3 | State specific objectives, including any prespecified hypotheses |  | Introduction, third paragraph |
| Methods | | |  |  |
| Study design | 4 | Present key elements of study design early in the paper |  | Methods, first section: Data sources |
| Setting | 5 | Describe the setting, locations, and relevant dates, including periods of recruitment, exposure, follow-up, and data collection |  | Methods, first section: Data sources |
| Participants | 6 | (*a*) Give the eligibility criteria, and the sources and methods of selection of participants. Describe methods of follow-up |  | Methods, third section: Study Population |
|  |  | (*b*) For matched studies, give matching criteria and number of exposed and unexposed | not applicable | not applicable |
| Variables | 7 | Clearly define all outcomes, exposures, predictors, potential confounders, and effect modifiers. Give diagnostic criteria, if applicable |  | Methods: second section: Exposures, outcomes and covariates |
| Data sources/ measurement | 8* | For each variable of interest, give sources of data and details of methods of assessment (measurement). Describe comparability of assessment methods if there is more than one group | p6-7 | Methods: second section: Exposures, outcomes and covariates |
| Bias | 9 | Describe any efforts to address potential sources of bias |  | Methods, fourth section: Statistical analysis, paragraph one and two |
| Study size | 10 | Explain how the study size was arrived at |  | Methods, third section: Study Population and figure 1 |
| Quantitative variables | 11 | Explain how quantitative variables were handled in the analyses. If applicable, describe which groupings were chosen and why |  | Methods: third section: Exposure, outcomes and covariates |
| Statistical methods | 12 | (*a*) Describe all statistical methods, including those used to control for confounding |  | Methods: fourth section: Statistical Analysis |
|  |  | (*b*) Describe any methods used to examine subgroups and interactions |  | Methods: fourth section: Statistical analysis: parahraph 2 |
|  |  | (*c*) Explain how missing data were addressed |  | Methods: third section: Study Poopulation and figure 1 |
|  |  | (*d*) If applicable, explain how loss to follow-up was addressed |  | Not applicable |
|  |  | (*e*) Describe any sensitivity analyses |  | Methods: fourth section: Statistical analysis, second paragraph |
| Results | | |  |  |
| Participants | 13* | (a) Report numbers of individuals at each stage of study—eg numbers potentially eligible, examined for eligibility, confirmed eligible, included in the study, completing follow-up, and analysed |  | Methods: third section: Study Population and figure 1 |
|  |  | (b) Give reasons for non-participation at each stage |  | Methods: third paragraph and figure 1 |
|  |  | (c) Consider use of a flow diagram |  | Figure 1 |
| Descriptive data | 14* | (a) Give characteristics of study participants (eg demographic, clinical, social) and information on exposures and potential confounders |  | Results: first section (Baseline Characteristics) and Table 1 |
|  |  | (b) Indicate number of participants with missing data for each variable of interest |  | Figure 1 |
|  |  | (c) Summarise follow-up time (eg, average and total amount) |  | Not applicable |
| Outcome data | 15* | Report numbers of outcome events or summary measures over time |  | Table 1 |
| Main results | 16 | (*a*) Give unadjusted estimates and, if applicable, confounder-adjusted estimates and their precision (eg, 95% confidence interval). Make clear which confounders were adjusted for and why they were included |  | Results: Section 2 (Birthweight) and 3 (Gestational age), Table 2 and 3, Figure 3, Supplementary table 3 and 4 |
|  |  | (*b*) Report category boundaries when continuous variables were categorized |  | Methods: second section: Exposure, outcomes, covariates, paragraph 3 |
|  |  | (*c*) If relevant, consider translating estimates of relative risk into absolute risk for a meaningful time period |  |  |
| Other analyses | 17 | Report other analyses done—eg analyses of subgroups and interactions, and sensitivity analyses |  | Results: Section 5: Sub-analysis, supplementary tables 5-14 |
| Discussion | | |  |  |
| Key results | 18 | Summarise key results with reference to study objectives | p14 | Discussion: Section 1: Summary of findings |
| Limitations | 19 | Discuss limitations of the study, taking into account sources of potential bias or imprecision. Discuss both direction and magnitude of any potential bias | p14-15 | Discussion: Section 2: Strengths and limitations |
| Interpretation | 20 | Give a cautious overall interpretation of results considering objectives, limitations, multiplicity of analyses, results from similar studies, and other relevant evidence | p16-17 | Discussion: Section 2: Strenghts and limitations and 3: Comparison with other studies |
| Generalisability | 21 | Discuss the generalisability (external validity) of the study results | p16 | Discussion: Section 2:Strenghts and limitations, last paragraph |
| Other information | | |  |  |
| Funding | 22 | Give the source of funding and the role of the funders for the present study and, if applicable, for the original study on which the present article is based | p18-19 | Footnotes: Funding |

*Give information separately for exposed and unexposed groups.

**Note:** An Explanation and Elaboration article discusses each checklist item and gives methodological background and published examples of transparent reporting. The STROBE checklist is best used in conjunction with this article (freely available on the Web sites of PLoS Medicine at http://www.plosmedicine.org/, Annals of Internal Medicine at http://www.annals.org/, and Epidemiology at http://www.epidem.com/). Information on the STROBE Initiative is available at http://www.strobe-statement.org.
