# Supplementary material for: Separating parental and treatment contributions to perinatal health after fresh and frozen embryo transfer in assisted reproduction: A cohort study with within-sibship analysis
Source: PLoS Med. 2021 Jun 25;18(6):e1003683. doi: 10.1371/journal.pmed.1003683 (PMC8274923; doi:10.1371/journal.pmed.1003683)
Supplement: S1 Study Protocol — (PDF) [file pmed.1003683.s002.pdf]

Cryopreservation of human embryos in assisted reproduction – consequences for perinatal health

Introduction

Over the last decades, assisted reproductive technology (ART) has become increasingly more common, due to a combination of increasing availability and success rates of treatment[1], but also societal changes with postponement of parenthood to age ranges with a low natural fertility[2]. Worldwide more than five million children have been born after assisted reproductive technology and in Europe more than 140 000 children are born after ART every year.[1,3] Today, one in six couples experience some form of infertility problem.[4] The Nordic countries have the highest availability of ART treatment in Europe, resulting in more than 10 000 children (3-6% of the birth cohorts) conceived each year following ART[1].

ART comprises all methods and techniques where fertilization occurs outside the female body and embryos are returned to the uterus, including standard in vitro fertilization (IVF) and intracytoplasmic sperm injection (ICSI), which is a more advanced method used for very poor semen quality. Prior to fertilization, controlled ovarian hyperstimulation is used to retrieve a high number of mature oocytes, through administration of hormones such as gonadotrophin-releasing hormone agonists and antagonists, follicle stimulating hormone, luteinizing hormone and human chorionic gonadotrophin[5]. In addition, progesterone is often given after fertilization and embryo transfer to improve chances of implantation[5]. In stimulated cycles, oestradiol levels can reach >4000 pg/mL[6], compared to 100-300 pg/mL in natural ovulatory cycles[7]. Furthermore, when treatment succeeds and implantation occurs, oestradiol levels remain elevated during the first trimester compared to naturally conceived pregnancies[8].

ART pregnancies carry a higher risk of adverse perinatal outcomes such as preterm birth, low or high birth weight, intrauterine or neonatal death, as well as congenital malformations.[9-11] The occurrence of pregnancy complications with potentially severe health consequences for both the mother and the foetus, including placental disorders and gestational diabetes, is also higher in ART compared to naturally conceived pregnancies.[11,12] A large proportion of the excess risk may be attributed to the high occurrence of twins and higher-order multiple pregnancies following ART conceptions.[13] However, ART singletons also carry a higher risk of adverse perinatal outcomes compared to naturally conceived singletons, but it is not known to what extent this may be direct effects of technology or treatment factors.[14]

A recurrent methodological challenge when studying adverse outcomes in ART pregnancies involves disentangling the potential contributions from the underlying physiology and health of infertile couples from potential adverse effects of the fertility treatment itself, including ovarian stimulation and laboratory methods (e.g. fertilization methods, culture conditions and culture duration).[15-17] Conditions associated with fertility problems, such as ovulation disorders, tubal factor, endometriosis and reduced sperm quality, are also associated with increased risk of adverse perinatal outcomes also following natural conceptions, and regardless of whether the couple has sought fertility treatment.[18] However, the severity of the conditions predisposing to infertility may well differ between couples who conceive with ART and those who eventually conceive naturally, and the two groups may therefore not be fully comparable. To investigate potential effects of ART treatment, comparison of sibling pregnancies may be applied to limit the influence of parental factors.[19] Such studies have been conducted previously using data from Nordic health registries and indicate that for the same mother, ART singletons have a higher risk of preterm birth than their naturally conceived siblings.[14,20,21] Contributions from the ART treatment itself could result from

both alterations in placental function or hormonal environment after ovarian stimulation, and from epigenetic or other changes in the embryo following laboratory procedures.[14,22,23]

New ART methods and drug regimens are continuously being implemented; often motivated by success in terms of live birth rates, rather than documentation of lower morbidity.[5,24] In consequence, little is known about safety aspects of more recent methods compared to standard IVF. One exception is the increasing use of elective single embryo transfer combined with cryopreservation (freezing) of excess embryos for later thawing and transfer, which has led to a decline in multiple pregnancies after ART over the last decade down to 18% in Europe.[1] Furthermore, an observed lower risk of preterm birth and low birthweight following cryopreservation compared to fresh ART has been attributed to the more physiological hormone levels in frozen-thawed cycles compared to fresh cycles, and abandoning fresh embryo transfer to the benefit of the "freeze-all policy" is currently debated.[25,26] It is therefore of great concern that several recent studies show that children born after frozen-thawed cycles are at increased risk of being born large for gestational age (LGA)[27,28], and that the risk of preeclampsia and gestational diabetes may also be higher than in pregnancies from fresh ART cycles.[28,29] The mechanisms behind these observations are currently completely unknown. In particular, it seems unlikely that known risk factors for LGA births in the general population such as maternal overweight and pre-existing diabetes mellitus[30,31], should differ substantially between women who conceive after fresh versus frozen-thawed ART cycles.[32] In pregnancies with foetal macrosomia or LGA outcome, there is an increased risk of stillbirth, foetal hypoxia, neonatal hypoglycaemia, maternal post-partum bleeding, as well as mechanical trauma for mother and child.[30] In a long-term perspective, high birthweight is associated with a higher risk of overweight and obesity in adulthood.[33,34]

Children born after cryopreservation now comprise more than 20-30% of all children born after ART in the Nordic countries.[1] In light of the increasing use of ART in general, the pivotal role of cryopreservation for prevention of multiple pregnancies, and the potential shift towards a "freeze-all policy", an improved understanding of the safety and risks associated with cryopreservation is urgently needed. Such knowledge may open opportunities for prevention of adverse consequences, either by changes in treatment practice or by interventions during pregnancy.

We therefore propose to study perinatal health following cryopreservation of embryos, focusing on understanding the association between cryopreservation and high birthweight. The proposed project will in part be based on a larger Nordic collaboration, The Committee of Nordic ART and Safety (CoNARTaS), established in 2008 with an overall aim to study the short and long term health and safety aspects for both children and mothers following ART conception. We will also establish a national collaboration involving several ART clinics.

Skills development

The project will strengthen the research collaboration between the Women's Clinic at St. Olavs Hospital and the Department of Public Health and General Practice at NTNU. By bringing methodological and clinical research competence closer together, this may provide opportunities for future collaborations and integrated projects.

The project group will develop competence in evaluation of safety aspects and adverse health outcomes of medical procedures based on observational methods and registry data linkage. In particular, the collaboration with methodological experts in sibling designs may further increase our understanding of potential challenges related to this approach that may be useful also in other disciplines. For reproductive medicine, the project will help establish a practice of evaluating health outcomes of newly introduced treatments, which will be particularly important in light of the continuous development in laboratory procedures of assisted reproduction.

We believe that the national collaboration that will be established as part of this project will provide a basis for further research, and thus strengthen reproductive medicine in Norway as a scientific discipline.

Research questions and goals

The overall aim of the project is to study perinatal health following cryopreservation of embryos, focusing on understanding the association between cryopreservation and high birthweight. Research question 1) and 2) will be studied in a cohort based on nation-wide health registry data from Sweden, Denmark, Finland and Norway, whereas 3) will be studied after combining data from five ART clinics in Norway.

Specifically, we propose to study:

(A) Does the risk of adverse perinatal outcomes differ between siblings conceived following fresh and frozen-thawed ART cycles?

A previous study of birthweight in fresh and frozen-thawed ART siblings indicates that the higher birthweight after cryopreservation may not be attributed to maternal factors.[35] In studies of unrelated individuals, cryopreservation has been associated with lower risk of preterm birth and a higher risk of perinatal death than in fresh ART pregnancies.[27] We will compare birthweight, risk of preterm birth, and perinatal death following fresh and frozen-thawed ART cycles. Comparisons will be made between all ART pregnancies, and between pregnancies by the same mother (sibling comparison).

(B) Is cryopreservation associated with a higher risk of gestational diabetes mellitus (GDM), and if so – does this contribute to the higher birthweight in such pregnancies?

GDM and maternal hyperglycaemia are strongly related to high birthweight.[36] Previous studies have shown either no difference in risk[32], or a higher risk[28], in pregnancies after frozen-thawed ART cycles compared to fresh ART cycles. We will compare the risk of GDM in pregnancies following fresh and frozen-thawed ART cycles to the risk in naturally conceived pregnancies. Furthermore, we will investigate to what extent occurrence of GDM can explain differences in birthweight between these groups. We hypothesize that the risk of GDM is higher after frozen-thawed ART compared to both fresh ART and natural conception, and that this may in part explain the higher birthweight in these pregnancies.

(C) Can the high birthweight in pregnancies following frozen-thawed ART cycles be attributed to other treatment factors?

It has been proposed that the low birthweight and increased risk of preterm birth in pregnancies after fresh ART cycles result in part from supraphysiological levels of ovarian hormones after controlled ovarian stimulation, and that this makes the endometrium less receptive to embryo implantation, partly due to lack of synchrony between endometrial and embryonal development.[25] With cryopreservation, embryo transfer can be postponed to cycles with normal hormone levels, although a large proportion of these cycles include some form of hormonal substitution for endometrial preparation[37]. Furthermore, in pregnancies after oocyte donation, where the recipient does not undergo controlled ovarian stimulation, similar birthweight has been observed between fresh and frozen-thawed embryo transfers.[38,39] Collectively, these results indicate that the high birthweight seen after cryopreservation, may not result from the freeze-thaw process itself, but that other treatment factors such as administration of hormones around the time of implantation may be involved. We will compare birthweight in pregnancies following cryopreservation with embryo transfer in natural cycles and cycles with different hormonal substitution regimens.

We expect that the three studies described above will be completed during the project period. On a longer term, we would like to develop further the collaboration between the Norwegian ART clinics that we will establish as part of the current project.

Expected significance for the field of knowledge

The increasing importance of embryo cryopreservation in the field of reproductive medicine stresses the need for a thorough evaluation of the safety aspects of this method. Comparing pregnancies with different conception methods within the same mother provides a unique opportunity to hold the contribution from maternal underlying biology relatively constant and thus examine the relation of conception methods to adverse pregnancy outcomes more directly. Sibling data on ART are not readily available outside the Nordic countries due to lack of nationwide registries and difficulties in linking information on ART treatment to information on children and their mothers. The study period will cover the entire period that ART has been available in the Nordic countries and the study population will be one of the largest on ART pregnancies worldwide. Furthermore, we are not aware of any previous studies comparing perinatal outcomes by different hormonal regimens in frozen-thawed ART cycles. We therefore expect that the results from this project will be unique worldwide and of significant value for the field of reproductive medicine.

Plan for implementation

Study design, choice of methods and analyses

The rationale and challenges in using sibling comparisons

Sibling comparisons have been used since the mid-20th century in epidemiological studies, but the conceptual framework and areas of application are continuously developing.[19,40,41] The design takes advantage of the fact that siblings share stable aspects of their family environment as well as half their genome, and they are therefore expected to be more similar than two randomly sampled individuals in the population.[40] Sibling discordance studies, where one sibling has the exposure of interest and the other does not, are particularly suitable for studying the influence of prenatal exposures, and can reduce the potential for confounding from the in utero environment and genetic factors substantially.[40]

Although comparisons of pregnancies after different conception methods in unrelated women may also adjust for confounding from parental factors, such information can only be included in the analyses to the extent that it has been measured. Detailed information on suspected confounding factors, such as causes for infertility and lifestyle factors, are often not available. In the challenge of disentangling the parental and treatment contributions to adverse outcomes in ART pregnancies, sibling designs therefore offer a valuable alternative approach, since both *measured* and *unmeasured* confounding is expected to be smaller between pregnancies in the same woman. However, it has been demonstrated that non-shared factors may result in stronger confounding for sibling comparisons than in studies of unrelated individuals.[19] Such factors would therefore require particular attention in the analyses. In this regard, it is reassuring that the underlying maternal factors and causes for infertility may be even more constant between ART sibling pregnancies than in the comparisons with naturally conceived siblings. In particular, pregnancies after fresh and frozen-thawed ART cycles may often result from the same ovarian stimulation and thus share the same pre-conception environment.

Data sources and study population for research studies A and B

The national ART and Medical Birth Registries in the Nordic countries (Sweden, Norway, Finland and Denmark) form the basis for the CoNARTaS cohort. The personal identification number for every citizen in each of the Nordic countries facilitates individual information linkage between health registries and other national registries. Thus, mothers and children after ART can be followed through available nationwide health registries. The Nordic situation is so far unique since no other countries have similar opportunities for linking nationwide data on ART treatment, parents and children. For each woman, we will therefore have information on all births, including information on date of birth, conception method, pregnancy characteristics and complications, as well as information on each child.

The CoNARTaS cohort will include all ART and naturally conceived children born from the year each country started its ART registration until 2014, and their mothers. Inclusion of the entire background population will provide the opportunity to conduct sibling comparisons for women who have conceived both naturally and through ART. Data from the following national registries will be included to provide data on ART exposures, neonatal outcomes and pregnancy complications, pre-pregnancy maternal health and causes for infertility, medical drug use, as well as maternal education: ART Registry, Medical Birth Registry, National Hospital Discharge Registry, Drug Prescription Registry, and National Registries on Education.

Table 1. Overview of the study population (approximate numbers)

	**Sweden**	**Norway**	**Finland**	**Denmark**	**CoNARTaS**
Study period	1982-2014	1984-2014	1990-2014	1994-2014	1982-2014
ART children (n)	60 000	32 000	30 000	45 000	167 000
ART pregnancies (n)	53 000	27 000	26 000	39 000	145 000
ART mothers (n)	50 500	21 000	22 500	35 000	129 000
NC children (n)	3 371 000	1 791 000	1 503 000	1 314 000	7 979 000
NC pregnancies (n)	3 330 000	1 768 000	1 481 000	1 292 000	7 871 000
NC mothers (n)	1 700 000	903 000	754 000	660 000	4 017 000

Data sources and study population for study C

In each treatment cycle, ART clinics in Norway collect substantially more detailed information than what is reported to the Medical Birth Registry, including information on the couple (social background and medical history), type of treatment and hormonal stimulation/preparation, as well as outcomes of each treatment cycle (assessment of developing embryos, obtained pregnancy, miscarriages). We will link the information from these records to information from the Medical Birth Registry to obtain perinatal and obstetric outcomes for treatment cycles resulting in pregnancies with duration beyond 12 weeks.

All clinics included in the planned collaboration use LinneFiler, which is a software tool designed for cycle-based registration of fertility treatment. This will greatly facilitate the process of extracting and linking data. We plan to include all cycles registered from the time LinneFiler was introduced at each clinic until the end of 2017 (see Progress schedule and Ethics). We have estimated that the resulting cohort will include >5000 deliveries after cryopreservation, where about 30% will have had embryo transfer in a natural cycle and the remaining 70% will have had one of several hormonal substitution regimens.

Statistical analyses

We will use linear and logistic regression to assess the associations between conception methods and perinatal and maternal outcomes at a population level. In sibling comparisons, we will use multilevel regression models, with separate levels for children (or pregnancies) and mothers,

including maternal identity as a random intercept variable.[42] This approach will allow simultaneous quantification of the contribution from the conception method (*within* mothers) and the contribution from maternal factors (*between* mothers) to the unexplained variance in the associations. When studying to what extent occurrence of GDM may explain the a higher birthweight in pregnancies after frozen-thawed ART cycles, we will apply the recently expanded framework for mediation analysis.[43] Precision will be estimated by 95% confidence intervals. For communication and public health purposes, we will report the magnitude of the observed associations in absolute terms such as risks, risk differences and attributable proportions.

We will include potential confounders as model covariates based on prior substance knowledge using directed acyclic graphs.[44] Factors to consider in this context include maternal age, parity and birth order, time between pregnancies, education, body mass index, smoking, foetal sex, duration of infertility, number of embryos transferred and multiple gestations. We will also adjust for socioeconomic status as measured by maternal education.

The CoNARTaS cohort will be considerable larger than previous studies, and will comprise >20 000 pairs of ART singleton siblings and in total >23 000 ART singleton pregnancies after cryopreservation. Based on previous studies from the Nordic countries,[20,21,27,29,35] we estimate that among the siblings, >700 pairs will discordant for both cycle status (fresh versus frozen-thawed) and preterm birth, whereas >100 pairs will be discordant for perinatal death, resulting in 80% power to detect odds ratios of ≥1.3 and ≥2.2 for these two outcomes, respectively (A). For GDM, we estimate that differences in occurrence ≥0.005 may be detected with 80% power in groups of ≥19 000 pregnancies (B). For birthweight, we will have 80% power to detect differences of ≥100 grams for groups of ≥570 pregnancies in unrelated women (C) and ≥340 pairs of siblings (A).

Since the different registries in each country do not collect identical information, we will use subsamples of the pooled data to conduct sensitivity analyses that may further enlighten the mechanisms involved, although statistical power in these analyses will be limited compared to the total sample. The Medical Birth Registries in Norway and Sweden contain information on paternal identity and age, which will allow us to take the paternal contribution to the associations into account to some extent. The ART registries in Sweden and Denmark are cycle-based (as opposed to pregnancy-based), which allow inclusion of ART treatment history for each mother.

Organization and collaboration

The applicant, Kjersti Westvik-Johari, is a specialist in Obstetrics and Gynaecology at the Fertility Section at the Women's Clinic, St. Olavs Hospital. Her clinical background from reproductive medicine and experience from using LinneFiler will be essential for the quality of the proposed studies. Main supervisor will be Signe Opdahl, postdoctoral fellow at the Department of Public Health and Nursing (ISM), NTNU, and project leader for the Norwegian contribution in CoNARTaS. She has extensive knowledge in epidemiological methods and handling of large data sets, including data from the current CoNARTaS cohort. As a PhD student, the applicant will join the Epidemiology and Medical Statistics research group (ISM), which has epidemiological research using data from registries and population-based cohorts as its main activity. Liv Bente Romundstad (Medical Director at Spiren Fertility Clinic, Trondheim) will be co-supervisor. She has been involved in establishing the CoNARTaS project and has comprehensive knowledge in reproductive medicine, as well as experience from research on data from Norwegian health registries and sibling designs.

We have also established collaboration with Centre for Fertility and Health (CFH) at the Norwegian Institute of Public Health (NIPH), recently appointed Centre of Excellence by the Research Council of Norway. Specialist Director at NIPH and leader of CFH Siri Eldevik Håberg will be co-supervisor in the proposed PhD project. She has extensive experience in conducting

epidemiological studies using national registry data. The applicant will also be invited to courses and research meetings organised by CFH.

Collaborators at each of the involved Norwegian ART clinics are Øyvind Nytun, Senior Physician at St. Olavs Hospital, Siren Skrede, Senior Embryologist and Head of the IVF Unit and Laboratory Director at Haukeland University Hospital, Hans Ivar Hanevik, Senior Physician at Fertilitetsklinikken Sør in Porsgrunn and Leader of the Norwegian Society for Assisted Reproduction (NOFAB), and Mette Haug Stensen, Senior Embryologist and Head of Laboratory at Fertilitetssenteret in Oslo. They will all be involved in planning, data extraction and analyses for study 3) and contribute substance knowledge in reproduction medicine and specific experience of how LinneFiler is used by their respective clinics.

The CoNARTaS project was established by the European IVF Monitoring group in the European Society of Human Reproduction and Embryology, and international collaboration was therefore established from the very beginning.[45] The group has had a successful collaboration with six publications in peer-reviewed international journals so far,[9,10,27,29,45,46] and four manuscripts in process, based on the initially established CoNARTaS cohort including data until 2007. The members of the group meet at least twice a year to discuss ongoing studies and planning of applications for data and funding.

Each country is represented by experienced team leaders with clinical and/or epidemiological and statistical background. The Danish team is led by Prof. Anja Pinborg at Hvidovre Hospital, Copenhagen University Hospital. She is a specialist in obstetrics and gynaecology with extensive experience in clinical and epidemiological research in reproductive medicine. Prof. Christina Bergh at Sahlgrenska Academy, University of Gothenburg, leads the Swedish team. She is head of the Swedish quality registry of ART, and specialist in obstetrics and gynaecology with long clinical experience in reproductive medicine. The Finnish team is led by Prof. Mika Gissler at the National Institute for Health and Welfare, Helsinki, with degrees in statistics, economics and epidemiology. He has extensive experience in registry-based epidemiology on perinatal and reproductive health using data from all the Nordic countries.

We have also established collaboration with Professor Debbie A Lawlor at the School of Social and Community Medicine, University of Bristol, UK. She is an internationally recognised epidemiologist with particular interest in sibling designs and perinatal medicine. Her contribution to development of analytical strategies for the sibling comparisons will be very valuable.

Progress schedule and publishing plan

For the CoNARTaS project (studies A and B), data extraction and linkage will soon be completed. Hvidovre Hospital has established a data sharing solution in collaboration with Statistics Denmark. Anonymized data from Denmark, Finland and Norway are uploaded at a secure server administered by Statistics Denmark, and Swedish data will be ready by September 2017. Data management and statistical analyses will be carried out by the group members via remote access to the server. A more detailed description of the data sharing platform is provided in the Ethics section below.

Although the national health registries in the Nordic countries are comparable in terms of which information is collected, pooling individual level data from a large number of sources requires extensive work in terms of harmonization and standardization of data. In this process, we will draw on the experiences from the pooling of data for the current CoNARTaS cohort. The data sharing solution will facilitate this work, since it enables researchers from each of the Nordic countries to work together on the same server. We have planned workshops at Hvidovre Hospital in Copenhagen dedicated to data management during fall 2017 and we expect that the standardization and preparation of data needed for analyses in studies A and B will be completed in 2017.

For study C, which will be based on linkage of data from the ART clinics and from the Medical Birth Registry of Norway, we will start by creating a more detailed plan for data extraction

in spring 2018. We aim to send the application for the Regional Committee for Ethics in Medical Research (REK) before June 2018, and to apply for data linkage during fall 2018. Adding expected waiting time for the data linkage, we estimate that the linked data will be ready for analyses early fall 2019, leaving sufficient time (about 16 months) to conduct study C.

Each of the studies A, B and C will result in separate manuscripts submitted for publication and we plan that study A will be submitted by month 16, study B by month 24 and study C by month 32 of the project period.

Plan for dissemination and implementation of results

We aim to publish the results in high-impact international peer-reviewed journals. To reach as many researchers and clinicians as possible, open-access publication will be preferred, and potential journals include Human Reproduction, Fertility and Sterility and American Journal of Obstetrics & Gynaecology. The results will also be presented at conferences hosted by different international societies in reproductive medicine. When appropriate from a pedagogical point of view, the results will also be included as practical examples in teaching of medical students and master students in clinical health science. Reaching the scientific and clinical community, as well as the societies in reproductive medicine, will form the starting point for clinical implementation of the knowledge obtained in the project, and for communication with patients. The broad collaboration of national and Nordic experts in the field will facilitate this process.

Assisted reproduction frequently attracts the attention of the media and is often considered as controversial or sensitive. Moreover, news headlines on adverse outcomes in ART pregnancies may cause great concern for couples undergoing ART treatment. Still, reaching the general public is important to ensure informed decisions on family planning. A balanced and neutral communication of the results is therefore crucial. User involvement from the Norwegian society for people who experience involuntary childlessness, Ønskebarn, will ensure that the necessary attention is given to the concerns and needs for information among infertile couples during the dissemination process (described in more detail below). To prepare dissemination outside the scientific and clinical community, we will consult the Communication Division at NTNU whenever new results are accepted for publication. Presenting absolute measures of risk and using the combined clinical experience of the CoNARTaS members from communication with couples seeking fertility treatment, will be essential parts of the dissemination strategies. A website for the CoNARTaS collaboration is under development (www.conartas.com), where we will publish plain language summaries of the project results.

User participation

The key users of the information obtained in this project will be the scientific and clinical community, people who experience fertility problems or who consider to postpone starting a family, as well as health authorities and society at large.

We have established collaboration with the Norwegian organization Ønskebarn, an interest group for people who experience involuntary childlessness. Ønskebarn has appointed a contact person who will give advice in the dissemination of results to the general public. We will also write plain language summaries more specifically adapted to the information needs of infertile couples that can be distributed to the members of Ønskebarn and published at their web page. We will also attend seminars hosted by Ønskebarn to inform about the project and have discussions about the content. Since the project is using existing data, user involvement in the data collection process will not be relevant.

Ethics

The CoNARTaS project is based only on registry data and involves no personal communication or intervention. The approvals from the relevant authorities or ethical committees as required in each of the four countries are obtained. According to the national laws in Denmark and Finland, studies solely based on register data do not need ethical approval from the Scientific Ethical Committees. Permission was granted from the regional ethics committees in Norway (REK 2010/1909) and Sweden (Dnr 023-09, T431-09). For all included children, mothers and fathers, the unique personal PIN-codes will be encrypted and replaced with anonymized serial numbers before data are sent to the researchers. Hence, no directly person-identifiable data will be used.

For individual health data, data security is one of the major ethical concerns. The CoNARTaS server at Statistics Denmark can be accessed only by group members and requires a two-step login procedure with personal password in combination with a one-time password created by an electronic code device. Anonymized data can be uploaded directly, thus avoiding physical transport. Standard statistical software is available at the server and analyses can be conducted only while being logged in and working at the data platform. The researchers will not be able to extract data from the platform and store them at other locations. Thus, the data sharing solution prevents any uncontrolled spread of sensitive data.

For study C, we will apply to REK for approval to extract and link data from the ART clinics and the Medical Birth Registry. Two of the clinics routinely collect informed consents from the treated couples to use the registered information for research purposes, whereas for the remaining three clinics, we will apply for exemption from confidentiality to extract information from LinneFiler for research purposes.

References

1. Calhaz-Jorge C, de Geyter C, Kupka MS, et al. Assisted reproductive technology in Europe, 2012: results generated from European registers by ESHRE. *Hum Reprod.* 2016;31(8):1638-1652.
2. Schmidt L, Sobotka T, Bentzen JG, Nyboe Andersen A. Demographic and medical consequences of the postponement of parenthood. *Hum Reprod Update.* 2012;18(1):29-43.
3. Ishihara O, Adamson GD, Dyer S, et al. International committee for monitoring assisted reproductive technologies: world report on assisted reproductive technologies, 2007. *Fertil Steril.* 2015;103(2):402-413.e411.
4. ESHRE. ART fact sheet. 2014; https://www.eshre.eu/Guidelines-and-Legal/ART-fact-sheet.aspx. Accessed 19.05.2016.
5. Farquhar C, Rishworth JR, Brown J, Nelen WL, Marjoribanks J. Assisted reproductive technology: an overview of Cochrane Reviews. *The Cochrane database of systematic reviews.* 2015;7:Cd010537.
6. Joo BS, Park SH, An BM, Kim KS, Moon SE, Moon HS. Serum estradiol levels during controlled ovarian hyperstimulation influence the pregnancy outcome of in vitro fertilization in a concentration-dependent manner. *Fertil Steril.* 2010;93(2):442-446.
7. Khodr ZG, Sherman ME, Pfeiffer RM, et al. Circulating sex hormones and terminal duct lobular unit involution of the normal breast. *Cancer Epidemiol Biomarkers Prev.* 2014;23(12):2765-2773.
8. Hu XL, Feng C, Lin XH, et al. High maternal serum estradiol environment in the first trimester is associated with the increased risk of small-for-gestational-age birth. *J Clin Endocrinol Metab.* 2014;99(6):2217-2224.
9. Henningsen AA, Wennerholm UB, Gissler M, et al. Risk of stillbirth and infant deaths after assisted reproductive technology: a Nordic study from the CoNARTaS group. *Hum Reprod.* 2014;29(5):1090-1096.
10. Henningsen AA, Gissler M, Skjaerven R, et al. Trends in perinatal health after assisted reproduction: a Nordic study from the CoNARTaS group. *Hum Reprod.* 2015;30(3):710-716.
11. Qin J, Liu X, Sheng X, Wang H, Gao S. Assisted reproductive technology and the risk of pregnancy-related complications and adverse pregnancy outcomes in singleton pregnancies: a meta-analysis of cohort studies. *Fertil Steril.* 2016;105(1):73-85.e71-76.
12. Jackson RA, Gibson KA, Wu YW, Croughan MS. Perinatal outcomes in singletons following in vitro fertilization: a meta-analysis. *Obstet Gynecol.* 2004;103(3):551-563.
13. Qin J, Wang H, Sheng X, Liang D, Tan H, Xia J. Pregnancy-related complications and adverse pregnancy outcomes in multiple pregnancies resulting from assisted reproductive technology: a meta-analysis of cohort studies. *Fertil Steril.* 2015;103(6):1492-1508.e1491-1497.
14. Pinborg A, Wennerholm UB, Romundstad LB, et al. Why do singletons conceived after assisted reproduction technology have adverse perinatal outcome? Systematic review and meta-analysis. *Hum Reprod Update.* 2013;19(2):87-104.
15. Kleijkers SH, Mantikou E, Slappendel E, et al. Influence of embryo culture medium (G5 and HTF) on pregnancy and perinatal outcome after IVF: a multicenter RCT. *Hum Reprod.* 2016;31(10):2219-2230.

16. Pereira N, Elias RT, Christos PJ, et al. Supraphysiologic estradiol is an independent predictor of low birth weight in full-term singletons born after fresh embryo transfer. *Hum Reprod.* 2017:1-8.
17. Sunkara SK, LaMarca A, Polyzos NP, Seed PT, Khalaf Y. Live birth and perinatal outcomes following stimulated and unstimulated IVF: analysis of over two decades of a nationwide data. *Hum Reprod.* 2016;31(10):2261-2267.
18. Stern JE, Luke B, Tobias M, Gopal D, Hornstein MD, Diop H. Adverse pregnancy and birth outcomes associated with underlying diagnosis with and without assisted reproductive technology treatment. *Fertil Steril.* 2015;103(6):1438-1445.
19. Frisell T, Oberg S, Kuja-Halkola R, Sjolander A. Sibling comparison designs: bias from non-shared confounders and measurement error. *Epidemiology.* 2012;23(5):713-720.
20. Romundstad LB, Romundstad PR, Sunde A, et al. Effects of technology or maternal factors on perinatal outcome after assisted fertilisation: a population-based cohort study. *Lancet.* 2008;372(9640):737-743.
21. Henningsen AK, Pinborg A, Lidegaard O, Vestergaard C, Forman JL, Andersen AN. Perinatal outcome of singleton siblings born after assisted reproductive technology and spontaneous conception: Danish national sibling-cohort study. *Fertil Steril.* 2011;95(3):959-963.
22. Choux C, Carmignac V, Bruno C, Sagot P, Vaiman D, Fauque P. The placenta: phenotypic and epigenetic modifications induced by Assisted Reproductive Technologies throughout pregnancy. *Clinical epigenetics.* 2015;7(1):87.
23. Thomopoulos C, Tsioufis C, Michalopoulou H, Makris T, Papademetriou V, Stefanadis C. Assisted reproductive technology and pregnancy-related hypertensive complications: a systematic review. *J Hum Hypertens.* 2013;27(3):148-157.
24. Romundstad LB, Opdahl S, Pinborg A. Which treatment option for couples with unexplained or mild male subfertility? *BMJ.* 2015;350:g7843.
25. Shapiro BS, Daneshmand ST, Garner FC, Aguirre M, Hudson C. Clinical rationale for cryopreservation of entire embryo cohorts in lieu of fresh transfer. *Fertil Steril.* 2014;102(1):3-9.
26. Weinerman R, Mainigi M. Why we should transfer frozen instead of fresh embryos: the translational rationale. *Fertil Steril.* 2014;102(1):10-18.
27. Wennerholm UB, Henningsen AK, Romundstad LB, et al. Perinatal outcomes of children born after frozen-thawed embryo transfer: a Nordic cohort study from the CoNARTaS group. *Hum Reprod.* 2013;28(9):2545-2553.
28. Shavit T, Oron G, Weon-Young S, Holzer H, Tulandi T. Vitrified-warmed single-embryo transfers may be associated with increased maternal complications compared with fresh single-embryo transfers. *Reproductive biomedicine online.* 2017.
29. Opdahl S, Henningsen AA, Tiitinen A, et al. Risk of hypertensive disorders in pregnancies following assisted reproductive technology: a cohort study from the CoNARTaS group. *Hum Reprod.* 2015;30(7):1724-1731.
30. Culliney KA, Parry GK, Brown J, Crowther CA. Regimens of fetal surveillance of suspected large-for-gestational-age fetuses for improving health outcomes. *The Cochrane database of systematic reviews.* 2016;4:Cd011739.
31. Berger H, Gagnon R, Sermer M, et al. Diabetes in Pregnancy. *Journal of obstetrics and gynaecology Canada : JOGC = Journal d'obstetrique et gynecologie du Canada : JOGC.* 2016;38(7):667-679.e661.
32. Sazonova A, Kallen K, Thurin-Kjellberg A, Wennerholm UB, Bergh C. Obstetric outcome in singletons after in vitro fertilization with cryopreserved/thawed embryos. *Hum Reprod.* 2012;27(5):1343-1350.
33. Zhao Y, Wang SF, Mu M, Sheng J. Birth weight and overweight/obesity in adults: a meta-analysis. *Eur J Pediatr.* 2012;171(12):1737-1746.
34. Cnattingius S, Villamor E, Lagerros YT, Wikstrom AK, Granath F. High birth weight and obesity--a vicious circle across generations. *International journal of obesity (2005).* 2012;36(10):1320-1324.
35. Pinborg A, Henningsen AA, Loft A, Malchau SS, Forman J, Andersen AN. Large baby syndrome in singletons born after frozen embryo transfer (FET): is it due to maternal factors or the cryotechnique? *Hum Reprod.* 2014;29(3):618-627.
36. Farrar D, Simmonds M, Bryant M, et al. Hyperglycaemia and risk of adverse perinatal outcomes: systematic review and meta-analysis. *BMJ.* 2016;354:i4694.
37. Groenewoud ER, Cohlen BJ, Al-Oraiby A, et al. A randomized controlled, non-inferiority trial of modified natural versus artificial cycle for cryo-thawed embryo transfer. *Hum Reprod.* 2016;31(7):1483-1492.
38. Vidal M, Vellve K, Gonzalez-Comadran M, et al. Perinatal outcomes in children born after fresh or frozen embryo transfer: a Catalan cohort study based on 14,262 newborns. *Fertil Steril.* 2017;107(4):940-947.
39. Kalra SK, Ratcliffe SJ, Coutifaris C, Molinaro T, Barnhart KT. Ovarian stimulation and low birth weight in newborns conceived through in vitro fertilization. *Obstet Gynecol.* 2011;118(4):863-871.
40. Donovan SJ, Susser E. Commentary: Advent of sibling designs. *Int J Epidemiol.* 2011;40(2):345-349.
41. Keyes KM, Smith GD, Susser E. On sibling designs. *Epidemiology.* 2013;24(3):473-474.
42. Thoresen M, Gjessing HK. Mixed models. In: Veierød MB, Lydersen S, Laake P, eds. *Medical statistics in clinical and epidemiological research*. 1 ed. Oslo, Norway: Gyldendal akademisk; 2012.
43. VanderWeele TJ. Mediation Analysis: A Practitioner's Guide. *Annu Rev Public Health.* 2016;37:17-32.
44. Hernan MA, Robins JM. Instruments for causal inference: an epidemiologist's dream? *Epidemiology.* 2006;17(4):360-372.
45. Henningsen AK, Romundstad LB, Gissler M, et al. Infant and maternal health monitoring using a combined Nordic database on ART and safety. *Acta Obstet Gynecol Scand.* 2011;90(7):683-691.
46. Sundh KJ, Henningsen AK, Kallen K, et al. Cancer in children and young adults born after assisted reproductive technology: a Nordic cohort study from the Committee of Nordic ART and Safety (CoNARTaS). *Hum Reprod.* 2014.
