# Supplementary material for: Separating parental and treatment contributions to perinatal health after fresh and frozen embryo transfer in assisted reproduction: A cohort study with within-sibship analysis
Source: PLoS Med. 2021 Jun 25;18(6):e1003683. doi: 10.1371/journal.pmed.1003683 (PMC8274923; doi:10.1371/journal.pmed.1003683)
Supplement: S1 Text — Tables of main (Tables A and B) and sensitivity analyses (Tables C–L), and summary of previous sibship studies (Table M). (DOCX) [file pmed.1003683.s003.docx]

**SUPPORTING INFORMATION**

| **Table A. Risk of adverse neonatal outcomes by conception method: population estimates and within sibship estimates in Main Sample 1 (minimizing selection).** | | | | | | | | | | | | | | |
| --- | --- | --- | --- | --- | --- | --- | --- | --- | --- | --- | --- | --- | --- | --- |
| **Main Sample1** | **Population estimates (Random effects)** | | | | | | | |  | **Within sibship estimates (Fixed effects)** | | | | |
|  | **Numbers** | **Risk ^1^, %** | **RD ^1^, *pp*** | **Adj. RD (95% CI) ^2^** | | **OR ^1^** | **Adj. OR (95% CI) ^2^** | |  | **Numbers ^3^** | **Risk ^1^, %** | **OR ^1^** | **Adj. OR (95% CI) ^2^** | |
| **SGA** |  |  |  |  |  |  |  |  |  |  |  |  |  |  |
| Natural Conception | 4 414 703 | 3.6 | 0 | 0 | Ref. | 1 | 1 | Ref. |  | 33 889 | 3.0 | 1 | 1 | Ref. |
| Fresh-ET | 78 095 | 5.3 | 1.6 | 0.76 | (0.61 to 0.92) | 1.60 | 1.24 | (1.19 to 1.29) |  | 30 167 | 4.5 | 1.64 | 1.20 | (1.08 to 1.34) |
| Frozen-ET | 17 990 | 2.9 | -0.8 | -0.66 | (-0.91 to 0.41) | 0.75 | 0.72 | (0.65 to 0.80) |  | 9 589 | 2.2 | 0.74 | 0.81 | (0.66 to 1.00) |
| **LGA** |  |  |  |  |  |  |  |  |  |  |  |  |  |  |
| Natural Conception | 4 414 703 | 4.5 | 0 | 0 | Ref. | 1 | 1 | Ref. |  | 33 889 | 5.3 | 1 | 1 | Ref. |
| Fresh-ET | 78 095 | 3.6 | -0,9 | -0.14 | (-0.30 to 0.05) | 0.75 | 1.01 | (0.96 to 1.06) |  | 30 167 | 3.9 | 0.65 | 0.92 | (0.84 to 1.02) |
| Frozen-ET | 17 990 | 6.5 | 2.1 | 2.4 | (2.0 to 2.8) | 1.70 | 1.98 | (1.82 to 2.15) |  | 9 589 | 7.3 | 1.88 | 1.84 | (1.56 to 2.17) |
| **Preterm birth <37 weeks** | |  |  |  |  |  |  |  |  |  |  |  |  |  |
| Natural Conception | 4 414 703 | 4.8 |  | 0 | Ref. | 1 | 1 | Ref. |  | 33 889 | 5.3 | 1 | 1 | Ref. |
| Fresh-ET | 78 095 | 8.0 | 3.1 | 2.2 | (2.0 to 2.4) | 1.92 | 1.63 | (1.58 to 1.69) |  | 30 167 | 7.2 | 1.55 | 1.27 | (1.17 to 1.37) |
| Frozen-ET | 17 990 | 6.5 | 1.7 | 1.4 | (1.1 to 1.8) | 1.46 | 1.40 | (1.30 to 1.50) |  | 9 589 | 5.4 | 1.05 | 1.05 | (0.91 to 1.20) |
| **Preterm birth <32 weeks** | |  |  |  |  |  |  |  |  |  |  |  |  |  |
| Natural Conception | 4 414 703 | 0.6 | 0 | 0 | Ref. | 1 | 1 | Ref. |  | 33 889 | 0.9 | 1 | 1 | Ref. |
| Fresh-ET | 78 095 | 1.5 | 0.9 | 0.57 | (0.50 to 0.64) | 2.60 | 2.03 | (1.90 to 2.12) |  | 30 167 | 1.2 | 1.46 | 1.18 | (1.0 to 1.41) |
| Frozen-ET | 17 990 | 1.2 | 0.51 | 0.37 | (0.23 to 0.50) | 1.91 | 1.66 | (1.42 to 1.94) |  | 9 589 | 0.9 | 0.93 | 0.92 | (0.67 to 1.27) |

Abbreviations: Adj. – adjusted, CI – confidence interval, LGA – large for gestational age, OR – odds ratio, *pp* – percentage points, RD – risk difference, Ref. – reference, SGA – small for gestational age

^1^ Unadjusted. ^2^ Adjusted for maternal age, parity, year of birth. Random effects are additionally adjusted for country. ^3^ Numbers refer to children that are part of a sibling groups with at least two different conceptions methods within the group.

| **Table B. Risk of adverse neonatal outcomes by conception method: population estimates and within sibship estimates in Main Sample 2 (minimizing confounding).** | | | | | | | | | | | | | | |
| --- | --- | --- | --- | --- | --- | --- | --- | --- | --- | --- | --- | --- | --- | --- |
|  | **Population estimates (Random effects)** | | | | | | | |  | **Within sibship estimates (Fixed effects)** | | | | |
|  | **Numbers** | **Risk ^1^, %** | **RD ^1^, *pp*** | **Adj. RD (95% CI) ^2^** | | **OR ^1^** | **Adj. OR (95% CI) ^2^** | |  | **Numbers ^3^** | **Risk ^1^, %** | **OR ^1^** | **Adj. OR (95% CI) ^2^** | |
| **SGA** |  |  |  |  |  |  |  |  |  |  |  |  |  |  |
| Natural Conception | 2 548 239 | 3.3 | 0 | 0 | Ref. | 1 | 1 | Ref. |  | 19 656 | 2.5 | 1 | 1 | Ref. |
| Fresh-ET | 53 059 | 4.9 | 1.8 | 0.73 | (0.61 to 0.92) | 1.73 | 1.29 | (1.23 to 1.36) |  | 17 631 | 4.3 | 2.07 | 1.27 | (1.09 to 1.47) |
| Frozen-ET | 14 326 | 2.8 | -0.4 | -0.67 | (-0.91 to 0.41) | 0.87 | 0.75 | (0.67 to 0.85) |  | 6538 | 2.1 | 0.93 | 0.85 | (0.65 to 1.11) |
| **LGA** |  |  |  |  |  |  |  |  |  |  |  |  |  |  |
| Natural Conception | 2 548 239 | 4.4 | 0 | 0 | Ref. | 1 | 1 | Ref. |  | 19 656 | 5.4 | 1 | 1 | Ref. |
| Fresh-ET | 53 059 | 3.5 | -1.0 | -0.12 | (-0.30 to 0.07) | 0.72 | 0.96 | (0.90 to 1.02) |  | 17 631 | 3.7 | 0.59 | 0.92 | (0.80 to 1.05) |
| Frozen-ET | 14 326 | 6.3 | 1.9 | 2.4 | (2.0 to 2.8) | 1.65 | 1.90 | (1.73 to 2.09) |  | 6538 | 7.2 | 1.67 | 1.76 | (1.43 to 2.17) |
| **Preterm birth <37 weeks** | |  |  |  |  |  |  |  |  |  |  |  |  |  |
| Natural Conception | 2 548 239 | 4.5 | 0 | 0 | Ref. | 1 | 1 | Ref. |  | 19 656 | 4.7 | 1 | 1 | Ref. |
| Fresh-ET | 53 059 | 7.6 | 3.0 | 2.1 | (1.9 to 2.3) | 1.96 | 1.65 | (1.58 to 1.72) |  | 17 631 | 6.7 | 1.62 | 1.23 | (1.11 to 1.36) |
| Frozen-ET | 14 326 | 6.2 | 1.7 | 1.4 | (1.0 to 1.8) | 1.52 | 1.43 | (1.31 to 1.56) |  | 6538 | 5.2 | 1.21 | 1.20 | (1.00 to 1.44) |
| **Preterm birth <32 weeks** | |  |  |  |  |  |  |  |  |  |  |  |  |  |
| Natural Conception | 2 548 239 | 0.5 | 0 | 0 | Ref. | 1 | 1 | Ref. |  | 19 656 | 0.7 | 1 | 1 | Ref. |
| Fresh-ET | 53 059 | 1.3 | 0.76 | 0.50 | (0.41 to 0.57) | 2.67 | 2.05 | (1.87 to 2.25) |  | 17 631 | 1.0 | 1.36 | 1.05 | (0.81 to 1.35) |
| Frozen-ET | 14 326 | 1.0 | 0.45 | 0.32 | (0.17 to 0.47) | 1.96 | 1.66 | (1.37 to 2.00) |  | 6538 | 0.8 | 1.0 | 0.93 | (0.62 to 1.41) |

Abbreviations: Adj. – adjusted, CI – confidence interval, LGA – large for gestational age, OR – odds ratio, *pp* – percentage points, RD – risk difference, Ref. – reference, SGA – small for gestational age

^1^ Unadjusted. ^2^ Adjusted for maternal age, parity, year of birth, maternal pre-pregnancy or first trimester body mass index, maternal smoking during pregnancy. Random effects are additionally adjusted for country & maternal height. ^3^ Numbers refer to children that are part of a sibling groups with at least two different conceptions methods within the group.

| **Table C. Birthweight and gestational age by conception method: population estimates and within sibship estimates. Full siblings in Main Sample 1 (minimizing selection).** | | | | | | | | | | | |
| --- | --- | --- | --- | --- | --- | --- | --- | --- | --- | --- | --- |
|  | **Population estimates (Random effects)** | | | | |  | **Within sibship estimates (Fixed effects)** | | | | |
|  | **Numbers** | **Mean ^1^** | **Mean difference ^1^** | **Adj. mean difference (95% CI) ^2^** | |  | **Numbers ^3^** | **Mean ^1^** | **Mean difference ^1^** | **Adj. mean difference**  **(95% CI) ^2^** | |
| **Birthweight, grams** |  |  |  |  |  |  |  |  |  |  |  |
| Natural Conception | 3 390 496 | 3567 | 0 | 0 | Ref. |  | 30 639 | 3576 | 0 | 0 | Ref. |
| Fresh-ET | 40 765 | 3450 | -117 | -69 | (-74 to -64) |  | 27 318 | 3434 | -129 | -50 | (-57 to -43) |
| Frozen-ET | 10 699 | 3634 | 67 | 60 | (51 to 70) |  | 9 005 | 3630 | 67 | 78 | (66 to 90) |
| **Birthweight, z-score** |  |  |  |  |  |  |  |  |  |  |  |
| Natural Conception | 3 390 496 | 0.04 | 0 | 0 | Ref. |  | 30 639 | 0.07 | 0 | 0 | Ref. |
| Fresh-ET | 40 765 | -0.15 | -0.19 | -0.07 | (-0.08 to -0.06) |  | 27 318 | -0.24 | -0.27 | -0.07 | (-0.08 to -0.05) |
| Frozen-ET | 10 699 | 0.25 | 0.21 | 0.19 | (0.17 to 0.21) |  | 9 005 | 0.19 | 0.16 | 0.18 | (0.16 to 0.21) |
| **Gestational age, days** |  |  |  |  |  |  |  |  |  |  |  |
| Natural Conception | 3 390 496 | 279.3 | 0 | 0 | Ref. |  | 30 639 | 279.2 | 0 | 0 | Ref. |
| Fresh-ET | 40 765 | 277.5 | -1.8 | -1.8 | (-1.9 to -1.6) |  | 27 318 | 278.3 | -0.9 | -0.8 | (-1.0 to -0.6) |
| Frozen-ET | 10 699 | 278.6 | -0.6 | -0.6 | (-0.8 to -0.4) |  | 9 005 | 279.6 | 0.3 | 0.4 | (0.1 to 0.7) |

Abbreviations: Adj. – adjusted, CI – confidence interval, – reference

^1^ Unadjusted. ^2^ Adjusted for maternal age, parity, year of birth. Random effects are additionally adjusted for country. ^3^ Numbers refer to children that are part of a sibling groups with at least two different conceptions methods within the group.

| **Table D. Risk of adverse neonatal outcomes by conception method: population estimates and within sibship estimates. Full siblings in Main Sample 1 (minimizing selection).** | | | | | | | | | | | | | | |
| --- | --- | --- | --- | --- | --- | --- | --- | --- | --- | --- | --- | --- | --- | --- |
|  | **Population estimates (Random effects)** | | | | | | | |  | **Within sibship estimates (Fixed effects)** | | | | |
|  | **Numbers** | **Risk ^1^, %** | **RD ^1^, *pp*** | **Adj. RD (95% CI) ^2^** | | **OR ^1^** | **Adj. OR (95% CI) ^2^** | |  | **Numbers ^3^** | **Risk ^1^, %** | **OR ^1^** | **Adj. OR (95% CI) ^2^** | |
| **SGA** |  |  |  |  |  |  |  |  |  |  |  |  |  |  |
| Natural Conception | 3 390 496 | 3.1 | 0 | 0 | Ref. | 1 | 1 | Ref. |  | 30 639 | 2.7 | 1 | 1 | Ref. |
| Fresh-ET | 40 765 | 4.3 | 1.2 | 0.76 | (0.55 to 1.0) | 1.51 | 1.36 | (1.26 to 1.47) |  | 27 318 | 4.5 | 1.86 | 1.31 | (1.1 to 1.5) |
| Frozen-ET | 10 699 | 2.2 | -0.8 | -0.4 | (-0.77 to -0.12) | 0.70 | 0.80 | (0.68 to 0.96) |  | 9 005 | 2.3 | 0.86 | 0.91 | (0.7 to 1.2) |
| **LGA** |  |  |  |  |  |  |  |  |  |  |  |  |  |  |
| Natural Conception | 3 390 496 | 4.8 | 0 | 0 | Ref. | 1 | 1 | Ref. |  | 30 639 | 5.5 | 1 | 1 | Ref. |
| Fresh-ET | 40 765 | 4.0 | -1.0 | -0.30 | (-0.5 to 0.03) | 0.74 | 0.91 | (0.84 to 1.00) |  | 27 318 | 3.8 | 0.58 | 0.91 | (0.79 to 1.05) |
| Frozen-ET | 10 699 | 7.2 | 2.5 | 2.30 | (1.8 to 2.7) | 1.79 | 1.81 | (1.61 to 2.04) |  | 9 005 | 7.2 | 1.70 | 1.77 | (1.42 to 2.21) |
| **Preterm birth <37 weeks** | |  |  |  |  |  |  |  |  |  |  |  |  |  |
| Natural Conception | 3 390 496 | 4.4 | 0 | 0 | Ref. | 1 | 1 | Ref. |  | 30 639 | 5.0 | 1 | 1 | Ref. |
| Fresh-ET | 40 765 | 6.7 | 2.3 | 1.7 | (1.5 to 2.0) | 1.72 | 1.58 | (1.49 to 1.69) |  | 27 318 | 7.0 | 1.58 | 1.20 | (1.07 to 1.34) |
| Frozen-ET | 10 699 | 5.3 | 0.9 | 1.2 | (0.7 to 1.6) | 1.26 | 1.38 | (1.22 to 1.56) |  | 9 005 | 5.3 | 1.09 | 1.23 | (1.02 to 1.49) |
| **Preterm birth <32 weeks** | |  |  |  |  |  |  |  |  |  |  |  |  |  |
| Natural Conception | 3 390 496 | 0.5 | 0 | 0 | Ref. | 1 | 1 | Ref. |  | 30 639 | 0.8 | 1 | 1 | Ref. |
| Fresh-ET | 40 765 | 1.1 | 0.58 | 0.4 | (0.29 to 0.50) | 2.32 | 2.07 | (1.80 to 2.40) |  | 27 318 | 1.1 | 1.50 | 1.12 | (0.85 to 1.49) |
| Frozen-ET | 10 699 | 0.8 | 0.26 | 0.26 | (0.08 to 0.43) | 1.58 | 1.67 | (1.26 to 2.22) |  | 9 005 | 0.8 | 0.94 | 0.98 | (0.62 to 1.54) |

Abbreviations: Adj. – adjusted, CI – confidence interval, LGA – large for gestational age, OR – odds ratio, *pp* – percentage points, RD – risk difference, Ref. – reference, SGA – small for gestational age

^1^ Unadjusted. ^2^ Adjusted for maternal age, parity, year of birth. Random effects are additionally adjusted for country. ^3^ Numbers refer to children that are part of a sibling groups with at least two different conceptions methods within the group.

| **Table E. Birthweight and gestational age by conception method: population estimates and within sibship estimates. Restricted to participants with < 3-year interval between siblings in Main Sample 1 (minimizing selection).** | | | | | | | | | | | |
| --- | --- | --- | --- | --- | --- | --- | --- | --- | --- | --- | --- |
|  | **Population estimates (Random effects)** | | | | |  | **Within sibship estimates (Fixed effects)** | | | | |
|  | **Numbers ^1^** | **Mean ^2^** | **Mean difference ^2^** | **Adj. mean difference (95% CI) ^3^** | |  | **Numbers ^4^** | **Mean ^2^** | **Mean difference ^2^** | **Adj. mean difference**  **(95% CI) ^3^** | |
| **Birthweight, grams** |  |  |  |  |  |  |  |  |  |  |  |
| Natural Conception | 1 982 791 | 3557 | 0 | 0 | Ref. |  | 16 674 | 3558 | 0 | 0 | Ref. |
| Fresh-ET | 23 304 | 3426 | -131 | -63 | (-70 to 56) |  | 16 602 | 3378 | -180 | -38 | (-47 to -29) |
| Frozen-ET | 6 698 | 3621 | 64 | 67 | (55 to 79) |  | 5 580 | 3580 | 22 | 91 | (76 to 107) |
| **Birthweight, z-score** |  |  |  |  |  |  |  |  |  |  |  |
| Natural Conception | 1 982 791 | 0.024 | 0 | 0 | Ref. |  | 16 674 | 0.02 | 0 | 0 | Ref. |
| Fresh-ET | 23 304 | -0.23 | -0.25 | -0.06 | (-0.8 to -0.05) |  | 16 602 | -0.42 | -0.44 | -0.07 | (-0.09 to -0.05) |
| Frozen-ET | 6 698 | 0.2 | 0.18 | 0.20 | (0.18 to 0.22) |  | 5 580 | .03 | 0.01 | 0.19 | (0.16 to 0.22) |
| **Gestational age, days** |  |  |  |  |  |  |  |  |  |  |  |
| Natural Conception | 1 982 791 | 279.2 | 0 | 0 | Ref. |  | 16 674 | 279.2 | 0 | 0 | Ref. |
| Fresh-ET | 23 304 | 277.6 | -1.54 | -1.71 | (-1.87 to -1.55) |  | 16 602 | 278.9 | -0.28 | -0.43 | (-0.68 to -0.19) |
| Frozen-ET | 6 698 | 278.8 | -0.41 | -0.49 | (-0.78 to -0.20) |  | 5 580 | 280.0 | 0.84 | 0.72 | (0.31 to 1.13) |

Abbreviations: Adj. – adjusted, CI – confidence interval, Ref. – reference,

^1^ Unadjusted. ^2^ Adjusted for maternal age, parity, year of birth. Random effects are additionally adjusted for country. ^3^ Numbers refer to children that are part of a sibling groups with at least two different conceptions methods within the group.

| **Table F. Risk of adverse neonatal outcomes by conception method: population estimates and within sibship estimates. Restricted to participants with < 3-year interval between siblings from Main Sample 1 (minimizing selection).** | | | | | | | | | | | | | | |
| --- | --- | --- | --- | --- | --- | --- | --- | --- | --- | --- | --- | --- | --- | --- |
|  | **Population estimates (Random effects)** | | | | | | | |  | **Within sibship estimates (Fixed effects)** | | | | |
|  | **Numbers ^1^** | **Risk ^2^, %** | **RD ^2^, *pp*** | **Adj. RD (95% CI) ^3^** | | **OR ^2^** | **Adj. OR (95% CI) ^3^** | |  | **Numbers ^4^** | **Risk ^2^, %** | **OR ^2^** | **Adj. OR (95% CI) ^3^** | |
| **SGA** |  |  |  |  |  |  |  |  |  |  |  |  |  |  |
| Natural Conception | 1 982 791 | 3.1 | 0 | 0 | Ref. | 1 | 1 | Ref. |  | 16 674 | 2.3 | 1 | 1 | Ref. |
| Fresh-ET | 23 304 | 4.4 | 1.4 | 0.65 | (0.42 to 0.90) | 1.58 | 1.28 | (1.18 to 1.39) |  | 16 602 | 4.7 | 2.90 | 1.20 | (1.01 to 1.41) |
| Frozen-ET | 6 698 | 2.1 | -0.9 | -0.65 | (-1.04 to -0.25) | 0.66 | 0.75 | (0.61 to 0.91) |  | 5 580 | 2.2 | 1.30 | 0.89 | (0.66 to 1.20) |
| **LGA** |  |  |  |  |  |  |  |  |  |  |  |  |  |  |
| Natural Conception | 1 982 791 | 4.5 | 0 | 0 | Ref. | 1 | 1 | Ref. |  | 16 674 | 5.8 | 1 | 1 | Ref. |
| Fresh-ET | 23 304 | 3.7 | -1.1 | 0.01 | (-0.5 to 0.02) | 0.70 | 1.0 | (0.91 to 1.1) |  | 16 602 | 3.4 | 0.40 | 0.98 | (0.85 to 1.15) |
| Frozen-ET | 6 698 | 6.6 | 2.1 | 2.2 | (1.58 to 2.76) | 1.72 | 1.78 | (1.56 to 2.04) |  | 5 580 | 6.7 | 1.12 | 1.83 | (1.45 to 2.31) |
| **Preterm birth <37 weeks** | |  |  |  |  |  |  |  |  |  |  |  |  |  |
| Natural Conception | 1 982 791 | 4.4 | 0 | 0 | Ref. | 1 | 1 | Ref. |  | 16 674 | 4.6 | 1 | 1 | Ref. |
| Fresh-ET | 23 304 | 6.8 | 2.40 | 1.85 | (2.10 to 2.72) | 1.75 | 1.58 | (1.48 to 1.69) |  | 16 602 | 6.9 | 1.77 | 1.13 | (1.00 to 1.27) |
| Frozen-ET | 6 698 | 5.0 | 0.60 | 0.89 | (0.33 to 1.44) | 1.18 | 1.27 | (1.11 to 1.45) |  | 5 580 | 5.0 | 1.18 | 0.97 | (0.79 to 1.18) |
| **Preterm birth <32 weeks** | |  |  |  |  |  |  |  |  |  |  |  |  |  |
| Natural Conception | 1 982 791 | 0.5 | 0 | 0 | Ref. | 1 | 1 | Ref. |  | 16 674 | 0.9 | 1 | 1 | Ref. |
| Fresh-ET | 23 304 | 1.2 | 0.70 | 0.53 | (0.40 to 0.66) | 2.42 | 2.15 | (1.87 to 2.47) |  | 16 602 | 1.2 | 1.47 | 1.04 | (0.80 to 1.35) |
| Frozen-ET | 6 698 | 0.8 | 0.19 | 0.21 | (-0.004 to 0.41) | 1.40 | 1.42 | (1.04 to 1.94) |  | 5 580 | 0.8 | 0.88 | 0.78 | (0.48 to 1.27) |

Abbreviations: Adj. – adjusted, CI – confidence interval, LGA – large for gestational age, OR – odds ratio, *pp* – percentage points, RD – risk difference, Ref. – reference, SGA – small for gestational age

^1^ Unadjusted. ^2^ Adjusted for maternal age, parity, year of birth. Random effects are additionally adjusted for country. ^3^ Numbers refer to children that are part of a sibling groups with at least two different conceptions methods within the group.

| **Table G. Birthweight and gestational age by conception method: population estimates and within sibship estimates. Restricted to mothers with 2-4 children in Main Sample 1 (minimizing selection).** | | | | | | | | | | | |
| --- | --- | --- | --- | --- | --- | --- | --- | --- | --- | --- | --- |
|  | **Population estimates (Random effects)** | | | | |  | **Within sibship estimates (Fixed effects)** | | | | |
|  | **Numbers ^1^** | **Mean ^2^** | **Mean difference ^2^** | **Adj. mean difference (95% CI) ^3^** | |  | **Numbers ^4^** | **Mean ^2^** | **Mean difference ^2^** | **Adj. mean difference**  **(95% CI) ^3^** | |
| **Birthweight, grams** |  |  |  |  |  |  |  |  |  |  |  |
| Natural Conception | 3 694 258 | 3563 | 0 | 0 | Ref. |  | 33 889 | 3540 | 0 | 0 | Ref. |
| Fresh-ET | 44 019 | 3492 | -111 | -71 | (-76 to -66) |  | 30 167 | 3424 | -116.3 | -51 | (-58 to -45) |
| Frozen-ET | 11 321 | 3628 | 75 | 65 | (55 to 74) |  | 9 589 | 3623 | 83 | 82 | (70 to 94) |
| **Birthweight, z-score** |  |  |  |  |  |  |  |  |  |  |  |
| Natural Conception | 3 694 258 | 0.03 | 0 | 0 | Ref. |  | 33 889 | -0.01 | 0 | 0 | Ref. |
| Fresh-ET | 44 019 | -0.14 | -0.17 | -0.07 | (-0.08 to -0.06) |  | 30 167 | -0.23 | -0.22 | -0.06 | (-0.78 to -0.05) |
| Frozen-ET | 11 321 | 0.26 | 0.23 | 0.20 | (0.18 to 0.22) |  | 9 589 | 0.2 | 0.21 | 0.19 | (0.17 to 0.22) |
| **Gestational age, days** |  |  |  |  |  |  |  |  |  |  |  |
| Natural Conception | 3 694 258 | 279.2 | 0 | 0 | Ref. |  | 33 889 | 279.0 | 0 | 0 | Ref. |
| Fresh-ET | 44 019 | 277.3 | -1.90 | -1.86 | (-1.98 to -1.74) |  | 30 167 | 277.9 | -1.1 | -1.0 | (-1.2 to -0.84) |
| Frozen-ET | 11 321 | 278.6 | -0.62 | -0.49 | (-0.72 to -0.27) |  | 9 589 | 279.2 | 0.2 | 0.3 | (0.0 to 0.6) |

Abbreviations: Adj. – adjusted, CI – confidence interval, Ref. – reference

^1^ Unadjusted. ^2^ Adjusted for maternal age, parity, year of birth. Random effects are additionally adjusted for country. ^3^ Numbers refer to children that are part of a sibling groups with at least two different conceptions methods within the group.

| **Table H. Risk of adverse neonatal outcomes by conception method: population estimates and within sibship estimates. Restricted to mothers with 2-4 children in Main Sample 1 (minimizing selection).** | | | | | | | | | | | | | | |
| --- | --- | --- | --- | --- | --- | --- | --- | --- | --- | --- | --- | --- | --- | --- |
|  | **Population estimates (Random effects)** | | | | | | | |  | **Within sibship estimates (Fixed effects)** | | | | |
|  | **Numbers** | **Risk ^1^, %** | **RD ^2^, *pp*** | **Adj. RD (95% CI) ^2^** | | **OR ^1^** | **Adj. OR (95% CI) ^2^** | |  | **Numbers ^4^** | **Risk ^2^, %** | **OR ^2^** | **Adj. OR (95% CI) ^3^** | |
| **SGA** |  |  |  |  |  |  |  |  |  |  |  |  |  |  |
| Natural conception | 3 694 258 | 3.2 | 0 | 0 | Ref. | 1 | 1 | Ref. |  | 33 889 | 3.0 | 1 | 1 | Ref. |
| Fresh ET | 44 019 | 4.3 | 1.1 | 0.70 | (0.072 to 1.2) | 1.43 | 1.29 | (1.21 to 1.36) |  | 30 167 | 4.5 | 1.64 | 1.20 | (1.08 to 1.34) |
| Frozen ET | 11 321 | 2.2 | -1.0 | -0.70 | (-0.95 to -0.33) | 0.64 | 0.76 | (0.65 to 0.88) |  | 9 589 | 2.2 | 0.74 | 0.81 | (0.66 to 1.00) |
| **LGA** |  |  |  |  |  |  |  |  |  |  |  |  |  |  |
| Natural conception | 3 694 258 | 4.8 | 0 | 0 | Ref. | 1 | 1 | Ref. |  | 33 889 | 5.3 | 1 | 1 | Ref. |
| Fresh ET | 44 019 | 4.0 | -0.9 | 0.30 | (-0.50 to -0.03) | 0.74 | 0.91 | (0.84 to 0.99) |  | 30 167 | 3.9 | 0.65 | 0.92 | (0.84 to 1.02) |
| Frozen ET | 11 321 | 7.2 | 2.5 | 2.34 | (1.80 to 2.90) | 1.80 | 1.81 | (1.61 to 2.04) |  | 9 589 | 7.3 | 1.88 | 1.84 | (1.56 to 2.17) |
| **Preterm birth <37 weeks** | |  |  |  |  |  |  |  |  |  |  |  |  |  |
| Natural conception | 3 694 258 | 4.5 | 0 | 0 | Ref. | 1 | 1 | Ref. |  | 33 889 | 5.3 | 1 | 1 | Ref. |
| Fresh ET | 44 019 | 6.9 | 2.4 | 2.0 | (1.75 to 2.21) | 1.72 | 1.60 | (1.53 to 1.68) |  | 30 167 | 7.2 | 1.55 | 1.27 | (1.17 to 1.37) |
| Frozen ET | 11 321 | 5.3 | 0.8 | 1.0 | (0.60 to 1.56) | 1.22 | 1.30 | (1.18 to 1.44) |  | 9 589 | 5.4 | 1.05 | 1.05 | 0.91 to 1.20) |
| **Preterm birth <32 weeks** | |  |  |  |  |  |  |  |  |  |  |  |  |  |
| Natural conception | 3 694 258 | 0.56 | 0 | 0 | Ref. | 1 | 1 | Ref. |  | 33 889 | 0.9 | 1 | 1 | Ref. |
| Fresh ET | 44 019 | 1.21 | 0.63 | 0.52 | (0.43 to 0.63) | 2.31 | 2.10 | (1.90 to 2.32) |  | 30 167 | 1.2 | 1.46 | 1.18 | (1.0 to 1.41) |
| Frozen ET | 11 321 | 0.86 | 0.28 | 0.29 | (0.12 to 0.46) | 1.56 | 1.58 | (1.27 to 1.98) |  | 9 589 | 0.9 | 0.93 | 0.92 | (0.67 to 1.27) |

Abbreviations: Adj. – adjusted, CI – confidence interval, LGA – large for gestational age, OR – odds ratio, *pp* – percentage points, RD – risk difference, Ref. – reference, SGA – small for gestational age

^1^ Unadjusted. ^2^ Adjusted for maternal age, parity, year of birth. Random effects are additionally adjusted for country. ^3^ Numbers refer to children that are part of a sibling groups with at least two different conceptions methods within the group.

| **Table I. Birthweight and gestational age by conception method: population estimates and within sibship estimates. Children conceived by assisted reproduction are restricted to single embryo transfers in Main Sample 2 (minimizing confounding).** | | | | | | | | | | | |
| --- | --- | --- | --- | --- | --- | --- | --- | --- | --- | --- | --- |
|  | **Population estimates (Random effects)** | | | | |  | **Within sibship estimates (Fixed effects)** | | | | |
|  | **Numbers** | **Mean ^1^** | **Mean difference ^1^** | **Adj. mean difference (95% CI) ^2^** | |  | **Numbers ^3^** | **Mean ^1^** | **Mean difference ^1^** | **Adj. mean difference**  **(95% CI) ^2^** | |
| **Birthweight, grams** |  |  |  |  |  |  |  |  |  |  |  |
| Natural Conception | 2 548 239 | 3538 | 0 | 0 | Ref. |  | 19 656 | 3547 | 0 | 0 | Ref. |
| Fresh-ET | 29 606 | 3403 | -135 | -83 | (-89 to -77) |  | 10 684 | 3410 | -137 | -61 | (-72 to -49) |
| Frozen-ET | 9 850 | 3587 | 48 | 59 | (49 to 69) |  | 4 461 | 3622 | 76 | 74 | (56 to 92) |
| **Birthweight, z-score** |  |  |  |  |  |  |  |  |  |  |  |
| Natural Conception | 2 548 239 | -0.01 | 0 | 0 | Ref. |  | 19 656 | .007 | 0 | 0 | Ref. |
| Fresh-ET | 29 606 | -0.23 | -0.22 | -0.07 | (-0.09 to -0.06) |  | 10 684 | -0.27 | -0.27 | -0.08 | (-0.10 to 0.06) |
| Frozen-ET | 9 850 | 0.15 | 0.16 | 0.20 | (0.18 to 0.22) |  | 4 461 | -0.20 | 0.19 | 0.19 | (0.15 to 0.22) |
| **Gestational age, days** |  |  |  |  |  |  |  |  |  |  |  |
| Natural Conception | 2 548 239 | 279.0 | 0 | 0 | Ref. |  | 19 656 | 279.0 | 0 | 0 | Ref. |
| Fresh-ET | 29 606 | 277.0 | -2.0 | -2.1 | (-2.3 to 2.0) |  | 10 684 | 277.9 | -1.1 | -1.1 | (-1.4 to -0.9) |
| Frozen-ET | 9 850 | 278.4 | -0.6 | -0.7 | (-1.0 to -0.5) |  | 4 461 | 279.0 | 0.05 | 0.06 | (-0.4 to 0.5) |

Abbreviations: Adj. – adjusted, CI – confidence interval, LGA – large for gestational age, OR – odds ratio, *pp* – percentage points, RD – risk difference, Ref. – reference, SGA – small for gestational age.

^1^ Unadjusted. ^2^ Adjusted for maternal age, parity, year of birth, maternal pre-pregnancy or first trimester body mass index, maternal smoking during pregnancy. Random effects are additionally adjusted for country & maternal height. ^3^ Numbers refer to children that are part of a sibling groups with at least two different conceptions methods within the group.

| **Table J. Risk of adverse neonatal outcomes by conception method: population estimates and within sibship estimates. Children conceived by assisted reproduction are restricted to single embryo transfers in Main Sample 2 (minimizing confounding)** | | | | | | | | | | | | | | |
| --- | --- | --- | --- | --- | --- | --- | --- | --- | --- | --- | --- | --- | --- | --- |
|  | **Population estimates (Random effects)** | | | | | | | |  | **Within sibship estimates (Fixed effects)** | | | | |
|  | **Numbers** | **Risk ^1^, %** | **RD ^1^, *pp*** | **Adj. RD (95% CI) ^2^** | | **OR ^1^** | **Adj. OR (95% CI) ^2^** | |  | **Numbers ^3^** | **Risk ^1^, %** | **OR ^1^** | **Adj. OR (95% CI) ^2^** | |
| **SGA** |  |  |  |  |  |  |  |  |  |  |  |  |  |  |
| Natural Conception | 2 548 239 | 3.3 | 0 | 0 | Ref. | 1 | 1 | Ref. |  | 19 656 | 2.5 | 1 | 1 | Ref. |
| Fresh-ET | 29 606 | 4.9 | 1.67 | 0.70 | (0.50 to 0.90) | 1.67 | 1.28 | (1.20 to 1.37) |  | 10 684 | 4.2 | 2.24 | 1.44 | (1.18 to 1.76) |
| Frozen-ET | 9 850 | 2.8 | -0.50 | -0.73 | (-1.0 to -0.43) | 0.82 | 0.73 | (0.63 to 0.84) |  | 4 461 | 2.1 | 0.90 | 0.90 | (0.64 to 1.25) |
| **LGA** |  |  |  |  |  |  |  |  |  |  |  |  |  |  |
| Natural Conception | 2 548 239 | 4.4 | 0 | 0 | Ref. | 1 | 1 | Ref. |  | 19 656 | 5.4 | 1 | 1 | Ref. |
| Fresh-ET | 29 606 | 3.3 | -1.0 | -0.01 | (-0.27 to 0.24) | 0.71 | 1.0 | (0.92 to 1.08) |  | 10 684 | 3.8 | 0.63 | 0.97 | (0.81 to 1.15) |
| Frozen-ET | 9 850 | 6.4 | 2.1 | 2.6 | (2.05 to 3.05) | 1.72 | 1.96 | (1.75 to 2.20) |  | 4 461 | 7.5 | 1.93 | 2.05 | (1.57 to 2.66) |
| **Preterm birth <37 weeks** | |  |  |  |  |  |  |  |  |  |  |  |  |  |
| Natural Conception | 2 548 239 | 4.5 | 0 | 0 | Ref. | 1 | 1 | Ref. |  | 19 656 | 4.6 | 1 | 1 | Ref. |
| Fresh-ET | 29 606 | 7.4 | 2.90 | 2.08 | (1.80 to 2.35) | 1.91 | 1.64 | (1.55 to 1.74) |  | 10 684 | 6.6 | 1.75 | 1.35 | (1.17 to 1.57) |
| Frozen-ET | 9 850 | 6.1 | 1.57 | 1.37 | (0.91 to 1.83) | 1.46 | 1.41 | (1.27 to 1.56) |  | 4 461 | 4.9 | 1.18 | 1.22 | (0.97 to 1.54) |
| **Preterm birth <32 weeks** | |  |  |  |  |  |  |  |  |  |  |  |  |  |
| Natural Conception | 2 548 239 | 0.54 | 0 | 0 | Ref. | 1 | 1 | Ref. |  | 19 656 | 0.7 | 1 | 1 | Ref. |
| Fresh-ET | 29 606 | 1.2 | 0.70 | 0.46 | (0.35 to 0.56) | 2.52 | 2.00 | (1.76 to 2.25) |  | 10 684 | 0.9 | 1.20 | 0.94 | (0.67 to 1.32) |
| Frozen-ET | 9 850 | 0.96 | 0.43 | 0.30 | (0.13 to 0.47) | 1.91 | 1.63 | (1.29 to 2.05) |  | 4 461 | 0.7 | 0.90 | 0.89 | (0.52 to 1.51) |

**Abbreviations: Adj. – adjusted,** CI – confidence interval, LGA – large for gestational age, OR – odds ratio, *pp* – percentage points, RD – risk difference, Ref. – reference, SGA – small for gestational age

^1^ Unadjusted. ^2^ Adjusted for maternal age, parity, year of birth, maternal pre-pregnancy or first trimester body mass index, maternal smoking during pregnancy. Random effects are additionally adjusted for country & maternal height. ^3^ Numbers refer to children that are part of a sibling groups with at least two different conceptions methods within the group.

| **Table K. Birthweight and gestational age by conception method: population estimates and within sibship estimates. Children conceived by assisted reproduction are restricted to blastocyst transfers in Main Sample 2 (minimizing confounding).** | | | | | | | | | | | |
| --- | --- | --- | --- | --- | --- | --- | --- | --- | --- | --- | --- |
|  | **Population estimates (Random effects)** | | | | |  | **Within sibship estimates (Fixed effects)** | | | | |
|  | **Numbers ^1^** | **Mean ^2^** | **Mean difference ^2^** | **Adj. mean difference (95% CI) ^3^** | |  | **Numbers ^4^** | **Mean ^2^** | **Mean difference ^2^** | **Adj. mean difference**  **(95% CI) ^3^** | |
| **Birthweight, grams** |  |  |  |  |  |  |  |  |  |  |  |
| Natural Conception | 1 826 087 | 3544 | 0 | 0 | Ref. |  | 1696 | 3544 | 0 | 0 | Ref. |
| Fresh-ET | 4032 | 3430 | -110 | -77 | (-93 to -61) |  | 1190 | 3460 | -85 | -53 | (-85 to -22) |
| Frozen-ET | 3449 | 3593 | 57 | 81 | (63 to 98) |  | 880 | 3653 | 109 | 93 | (56 to 130) |
| **Birthweight, z-score** |  |  |  |  |  |  |  |  |  |  |  |
| Natural Conception | 1 826 087 | 0.004 | 0 | 0 | Ref. |  | 1696 | .004 | 0 | 0 | Ref. |
| Fresh-ET | 4032 | -0.12 | -0.13 | -0.03 | (-0.06 to -0.01) |  | 1190 | -.12 | -0.12 | -0.03 | (-0.10 to 0.03) |
| Frozen-ET | 3449 | 0.18 | 0.20 | 0.26 | (0.24 to 0.31) |  | 880 | .34 | 0.34 | 0.30 | (0.23 to 0.38) |
| **Gestational age, days** |  |  |  |  |  |  |  |  |  |  |  |
| Natural Conception | 1 826 087 | 278.9 | 0 | 0 | Ref. |  | 1696 | 278.9 | 0 | 0 | Ref. |
| Fresh-ET | 4032 | 276.4 | -2.40 | -2.68 | (-3.05 to -2.31) |  | 1190 | 277.4 | -1.49 | -1.58 | (-2.36 to -0.80) |
| Frozen-ET | 3449 | 278.2 | -0.68 | -0.95 | (-1.35 to -0.55) |  | 880 | 278.1 | -0.81 | -0.87 | (-1.78 to 0.5) |

Abbreviations: Adj. – adjusted, CI – confidence interval, Ref. – reference

^1^ Population restricted to singletons born from 1997 in Denmark, 2002 in Sweden and 2010 in Norway, when blastocyst transfer was implemented in fertility clinics in the respective countries. ^2^ Unadjusted. ^3^ Adjusted for maternal age, parity, year of birth, maternal pre-pregnancy or first trimester body mass index, maternal smoking during pregnancy. Random effects are additionally adjusted for country & maternal height.^4^ Numbers refer to children that are part of a full sibling group with at least two different conceptions methods within the group.

| **Table L. Risk of adverse neonatal outcomes by conception method: population estimates and within sibship estimates. Children conceived by assisted reproduction are restricted to blastocyst transfers in Main Sample 2 (minimizing confounding).** | | | | | | | | | | | | | | |
| --- | --- | --- | --- | --- | --- | --- | --- | --- | --- | --- | --- | --- | --- | --- |
|  | **Population estimates (Random effects)** | | | | | | | |  | **Within sibship estimates (Fixed effects)** | | | | |
|  | **Numbers ^1^** | **Risk ^2^, %** | **RD ^2^, *pp*** | **Adj. RD (95% CI) ^3^** | | **OR ^2^** | **Adj. OR (95% CI) ^3^** | |  | **Numbers ^4^** | **Risk ^2^, %** | **OR ^2^** | **Adj. OR (95% CI) ^3^** | |
| **SGA** |  |  |  |  |  |  |  |  |  |  |  |  |  |  |
| Natural Conception | 1 826 087 | 3.2 | 0 | 0 | Ref. | 1 | 1 | Ref. |  | 1696 | 2.5 | 1 | 1 | Ref. |
| Fresh-ET | 4032 | 4.5 | 1.29 | 0.62 | (0.072 to 1.2) | 1.52 | 1.26 | (1.04 to 1.52) |  | 1190 | 4.3 | 1.89 | 1.86 | (1.07 to 3.22) |
| Frozen-ET | 3449 | 2.6 | -0.72 | -1.07 | (-1.5 to -0.62) | 0.74 | 0.61 | (0.47 to 0.78) |  | 880 | 2.1 | 0.61 | 0.75 | (0.36 to 1.56) |
| **LGA** |  |  |  |  |  |  |  |  |  |  |  |  |  |  |
| Natural Conception | 1 826 087 | 4.4 | 0 | 0 | Ref. | 1 | 1 | Ref. |  | 1 696 | 4.7 | 1 | 1 | Ref. |
| Fresh-ET | 4032 | 3.9 | -0.47 | 0.49 | (-0.22 to 1.12) | 0.86 | 1.16 | (0.94 to 1.43) |  | 1190 | 4.5 | 1.03 | 1.43 | (0.90 to 2.30) |
| Frozen-ET | 3449 | 6.5 | 2.24 | 3.37 | (2.44 to 4.29) | 1.77 | 2.34 | (1.94 to 2.83) |  | 880 | 7.5 | 2.08 | 2.15 | (1.29 to 3.57) |
| **Preterm birth <37 weeks** | |  |  |  |  |  |  |  |  |  |  |  |  |  |
| Natural Conception | 1 826 087 | 4.5 | 0 | 0 | Ref. | 1 | 1 | Ref. |  | 1696 | 4.4 | 1 | 1 | Ref. |
| Fresh-ET | 4032 | 7.9 | 3.37 | 2.92 | (2.14 to 3.71) | 2.10 | 1.96 | (1.69 to 2.28) |  | 1190 | 6.6 | 1.66 | 1.65 | (1.01 to 2.47) |
| Frozen-ET | 3449 | 6.8 | 2.31 | 2.04 | (1.23 to 2.86) | 1.72 | 1.64 | (1.39 to 1.95) |  | 880 | 7.0 | 2.05 | 2.43 | (1.48 to 3.98) |
| **Preterm birth <32 weeks** | |  |  |  |  |  |  |  |  |  |  |  |  |  |
| Natural Conception | 1 826 087 | 0.6 | 0 | 0 | Ref. | 1 | 1 | Ref. |  | 1696 | 0.7 | 1 | 1 | Ref. |
| Fresh-ET | 4032 | 1.4 | 0.83 | 0.68 | (0.36 to 1.00) | 2.85 | 2.51 | (1.84 to 3.42) |  | 1190 | 1.1 | 1.70 | 1.58 | (0.62 to 4.02) |
| Frozen-ET | 3449 | 0.9 | 0.32 | 0.22 | (-0.06 to 0.50) | 1.67 | 1.47 | (0.97 to 2.21) |  | 880 | 0.7 | 1.17 | 1.29 | (0.38 to 4.43) |

Abbreviations: Adj. – adjusted, CI – confidence interval, LGA – large for gestational age, OR – odds ratio, *pp* – percentage points, RD – risk difference, Ref. – reference, SGA – small for gestational age

^1^ Population restricted to singletons born from 1997 in Denmark, 2002 in Sweden and 2010 in Norway, when blastocyst transfer was implemented in fertility clinics in the respective countries. ^2^ Unadjusted. ^3^ Adjusted for maternal age, parity, year of birth, maternal pre-pregnancy or first trimester body mass index, maternal smoking during pregnancy. Random effects are additionally adjusted for country & maternal height.^4^ Numbers refer to children that are part of a sibling group with at least two different conceptions methods within the group.

| **Table M. Overview and summary characteristics of previous sibling studies on perinatal health after assisted reproductive technology** | | | | | | | | |
| --- | --- | --- | --- | --- | --- | --- | --- | --- |
| **Study** | **Country** | **Study period** | **Comparison** | **Pairs (n)** | **Ref. level^1^** | **Unadj. estimate**  **(95% CI)** | **Adj. estimate**  **(95% CI)** | **Covariates** |
|  |  |  |  |  |  |  |  |  |
|  | | | |  |  | **Difference in mean birthweight, grams** | |  |
| **Romundstad et al 2008 ^2^** | Norway | 1988-2006 | ART vs NC | 2204 | 3538 | -87 (-125 to -49) | -9 (-36 to 18) | Gestational age, sex, maternal age, parity, birth year, pregnancy interval |
| **Seggers et al 2016 ^3^** | Nether-lands | 1999-2007 | ART vs NC | 1813 | 3467 | -105.0 (-146.0 to -62.8) | -25.3 (-29.4 to 77.8)^8^ | Sex, maternal age, parity, ethnicity, socioeconomic status, maternal diabetes, birth year, labour care |
| **Dhalwani et al 2016 ^4^** | USA | 2000-2010 | ART vs NC | 6458 | 3398 | -55.3 (-72.9 to -41.7) | -33.4 (-48.6 to -18.2) | Gestational age, sex, maternal age, parity, birth year, time since last delivery |
| **Goisis et al 2019 ^5^** | Finland | 1995-2000 | ART vs NC | 578 | 3594 | -137 (-189 to -85) | -31 (-85 to 22) | Sex, maternal age, parity, smoking, household income, multiple birth (incl. interaction with ART) |
| **Henningsen et al 2011^6^** | Denmark | 1994-2006 | Fresh-ET vs NC | 3879 | 3556 | -114 (-134 to -93) | -65 (-89 to -41) | Sex, maternal age, parity, and birth year |
|  |  |  | Frozen-ET vs Fresh-ET | 358 | 3443 | 202 (132 to 271) | 167 (90 to 244) |  |
| **Luke et al 2017 ^7^** | USA | 2004-2013 | Frozen-ET 2^nd^ vs Fresh-ET 1^st^ | 3371 | 3246 | 222 (200 to 244) | - | Unadjusted, but restricted to siblings with same sex |
|  |  |  | Frozen-ET 1^st^ vs Fresh-ET 2^nd^ | 310 | 3295 | 81 (8 to 154) | - |  |
|  |  |  |  |  |  |  |  |  |
|  | | | |  |  | **Difference in mean gestational age, days** | |  |
| **Romundstad et al 2008 ^2^** | Norway | 1988-2006 | ART vs NC | 2204 | 278.7 | -2.0 (-2.9 to -1.0) | -1.3 (-2.4 to -0.3) | Sex, maternal age, parity, birth year, pregnancy interval |
| **Seggers et al 2016 ^3^** | Nether-lands | 1999-2007 | ART vs NC | 1813 | 276 | -0.85 (-1.9 to 0.2) | -0.12 (-0.08 to 0.32)^8^ | Sex, maternal age, parity, ethnicity, socioeconomic status, maternal diabetes, birth year, labour care |
| **Dhalwani et al 2016 ^4^** | USA | 2000-2010 | ART vs NC | 6458 | 270.3 | -0.58 (-0.99 to -0.17) | -0.58 (-1.02 to -0.14) | Sex, maternal age, parity, birth year, time since last delivery |
| **Goisis et al 2019 ^5^** | Finland | 1995-2000 | ART vs NC | 578 | 278 | -2.5 (-3.7 to -1.2) | -1.3 (-2.6 to 0.0) | Sex, maternal age, parity, smoking, household income, multiple birth (incl. interaction with ART) |
| **Henningsen et al 2011^6^** | Denmark | 1994-2006 | Fresh-ET vs NC | 3879 | 277.2 | -0.6 (-1.1 to -0.1) | -1.4 (-2.0 to -0.7) | Sex, maternal age, parity, and birth year |
|  |  |  | Frozen-ET vs Fresh-ET | 358 | 277.6 | 0.2 (-1.5 to 1.9) | 1.5 (-0.3 to 3.3) |  |
| **Luke et al 2017 ^7^** | USA | 2004-2013 | Frozen-ET 2^nd^ vs Fresh-ET 1^st^ | 3371 | 267.7 | -0.3 (-0.9 to 0.3) | - | Unadjusted, but restricted to siblings with same sex |
|  |  |  | Frozen-ET 1^st^ vs Fresh-ET 2^nd^ | 310 | 266.2 | 2.9 (0.7 to 5.1) | - |  |

Abbreviations: Adj. – adjusted, ART – assisted reproductive technology, CI – confidence interval, ET – embryo transfer, Ref. – reference, Unadj. - unadjusted

^1^ Crude mean value in the reference category of the sibling group, i.e. the naturally conceived sibling in ART vs natural conception and the Fresh-ET sibling in Frozen-ET vs Fresh-ET.

^2^ Romundstad LB, Romundstad PR, Sunde A, von Düring V, Skjaerven R, Gunnell D, et al. Effects of technology or maternal factors on perinatal outcome after assisted fertilisation: a population-based cohort study. Lancet. 2008;372(9640):737-43. ^3^ Seggers J, Pontesilli M, Ravelli ACJ, Painter RC, Hadders-Algra M, Heineman MJ, et al. Effects of in vitro fertilization and maternal characteristics on perinatal outcomes: a population-based study using siblings. Fertil Steril. 2016;105(3):590-8. ^4^ Dhalwani NN, Boulet SL, Kissin DM, Zhang Y, McKane P, Bailey MA, et al. Assisted reproductive technology and perinatal outcomes: conventional versus discordant-sibling design. Fertil Steril. 2016;106(3):710-6. ^5^ Goisis A, Remes H, Martikainen P, Klemetti R, Myrskylä M. Medically assisted reproduction and birth outcomes: a within-family analysis using Finnish population registers. Lancet. 2019;393(10177):1225-32. ^6^ Henningsen AK, Pinborg A, Lidegaard Ø, Vestergaard C, Forman JL, Andersen AN. Perinatal outcome of singleton siblings born after assisted reproductive technology and spontaneous conception: Danish national sibling-cohort study. Fertil Steril. 2011;95(3):959-63. ^7^ Luke B, Brown MB, Wantman E, Stern JE, Toner JP, Coddington CC, 3rd. Increased risk of large-for-gestational age birthweight in singleton siblings conceived with in vitro fertilization in frozen versus fresh cycles. J Assist Reprod Genet. 2017;34(2):191-200. ^8^ Note: Asymmetry between point estimate and CI indicates probable error in point estimate and/or CI.
