# Supplementary material for: Separating parental and treatment contributions to perinatal health after fresh and frozen embryo transfer in assisted reproduction: A cohort study with within-sibship analysis
Source: PLoS Med. 2021 Jun 25;18(6):e1003683. doi: 10.1371/journal.pmed.1003683 (PMC8274923; doi:10.1371/journal.pmed.1003683)
Supplement: Attachment — Submitted filename: Responseletter_jan_again.docx [file pmed.1003683.s004.docx]

Editorial Office

Plos Medicine

Dear Editor,

We thank the reviewers and the editor for the valuable comments and the opportunity to improve and resubmit our work. Our responses are outlined in the attached document, first repeating the comments and with our responses in *italic, blue text*. We hope that you will find all comments to be adequately addressed.

Yours sincerely,

Kjersti Westvik-Johari

Requests from the editors:

1. To your data statement, please add (non-author) contact information for the study administrators or Statistics Denmark.

*We have added the following contact information to Statistics Denmark:*

*Division of Research Services*

*Statistics Denmark*

*Sejrøgade 11*

*DK-2100 Copenhagen*

*Denmark*

*E-mail:* [forskningsservice@dst.dk](mailto:forskningsservice@dst.dk)

*Phone: +45 39 17 31 30*

2. Considering the observational study design, please avoid language that implies causality (e.g., "effects" early in your abstract).

*We have adapted the language to avoid implying causality when describing the results of our study.*

3. Please adapt your abstract to a three-part structure - you may find it helpful to consult one or two recent research papers published in PLOS Medicine to get a sense of the preferred style.

*The abstract has been adapted to a three-part structure.*

4. The final sentence of the "Methods and findings" subsection of your abstract should begin "Study limitations include ..." or similar and quote 2-3 of the study's main limitations.

*We have included a sentence on the study’s limitations as requested.*

5. Please adapt the text to "We found ... had ..." towards the end of your abstract.

*The text has been adapted.*

6. After the abstract, please add an "Author summary" section in non-identical prose, aiming to use the active voice in 1-2 points (e.g., "We found that ...").

*The following Author summary has been added:*

***Author Summary:Why was this study done?***

- *Children born after assisted reproductive technology have more adverse perinatal outcomes than naturally conceived children, that differ according to treatment method.*
- *It is unknown to what extent these associations result from the fertility treatment or from confounding by underlying maternal or family factors.*

***What did the researchers do and find?***

- *Using health registry data from Denmark, Norway and Sweden, we compared perinatal health after conception by fresh or frozen embryo transfer to natural conception, in a cohort of 4 606 875 newborns. In addition, we compared siblings conceived by different methods to account for family confounding (n=33 056 sibling groups).*
- *We found that children of frozen-ET have a higher birthweights and risk of large for gestational age, whereas children of fresh-ET have a lower birthweights and higher risk of small for gestational compared to naturally conceived children, both in the population and within siblings.*
- *Within siblings, children of fresh-ET and frozen-ET had an increased risk of preterm birth (<37 weeks) of similar magnitude, while neither fresh- nor frozen-ET were associated with risk of very preterm birth (<32 weeks), despite strong associations for both outcomes in population analyses.*

***What do these findings mean?***

- *Fresh and frozen embryo transfer showed opposite associations with birthweight, but similar associations with preterm birth, after controlling for measured and unmeasured family level confounding.*
- *Both treatments are associated with adverse perinatal outcomes, in comparison to natural conception. Our findings provide important information that can be used by couples and their clinicians in making decisions about which type of ART to undertake.*

7. Early in the methods section of your main text, please state whether the study had a protocol or prespecified analysis plan, and if so attach the relevant document(s) as a supplementary file(s), referred to in the text. Please highlight analyses that were not prespecified.

*The study had a prespecified protocol from the study’s funding proposal ,uploaded as a supplementary file. The study protocol was followed with the exception that perinatal death, which was among the prespecified outcomes, will be published in a separate paper (ongoing work). This change was made when we realized that the large number of analyses and results would be difficult to describe and discuss sufficiently in a single paper. We have included the following sentence in the Methods section, page 11, in the last paragraph of Statistical Analysis:*

*The study protocol was specified prior to data analysis (S2_Study_protocol) and the remaining study questions is the topic of ongoing work.*

8. Throughout the text, please adapt reference call-outs to the following style: "... socioeconomic position [16,17]." (i.e., with no spaces within the square brackets).

*The journal’s reference style has been adapted.*

9. Throughout the paper, please quote p values alongside 95% CI where available.

*We have added p-values alongside the 95% CIs in the abstract.*

10. Please ensure that all reference citations meet journal style. Six author names should be listed rather than 3, followed by "et al."; italics should be converted to plain text.

*The journal’s reference style has been adapted.*

11. add a completed checklist for the most appropriate reporting guideline - which may be STROBE or RECORD - as a supplementary document. Please refer to this in the methods section of your main text (e.g., "See S1_RECORD_Checklist"). In the checklist, please refer to individual items by section (e.g., "Methods") and paragraph number rather than by line or page numbers, as the latter generally change in the event of publication.

*A completed STROBE checklist for cohort studies is included as a supplementary document S1 STROBE Checklist.*

Comments from the reviewers:

*** Reviewer #1:

"Perinatal health after fresh and frozen embryo transfer in assisted reproduction - separating parental and treatment contributions. A cohort study with within sib-ship analysis" examines the correlations between fresh embryo transfer (fresh-ET) and frozen embryo transfer (frozen-ET), to various outcomes such as birthweight and preterm birth. Over 33,000 discordant sibling pairs from three countries were included, with two main analyses performed to minimize selection bias and minimize confounding, respectively. Various sensitivity analyses were also performed to study the effect of additional potential confounders. It was consistently found that infants conceived from fresh-ET tend to have lower birthweight/being small for gestational age, while those conceived from frozen-ET tend to have higher birthweight/being large for gestation age. Both fresh-ET and frozen-ET had increased risk of preterm birth. These findings largely concur with prior literature, which however did not generally account for confoundings as thoroughly.

The analyses appear convincing and robust on the whole, particularly as the sibship constraint helps to mitigate a large number of potential confounders, due to sharing the same parent(s).

1. It is stated that small for gestational age (SGA) was defined as birthweights less than 2 std dev, and large for gestational age (LGA) as birthweights greater than 2 std dev, where one standard deviation was set as 11% of the expected birthweight. However, it appears that the most common definition would be a weight below 10th percentile for SGA (and conversely above 90th percentile for LGA). Was there any reason as to why the 2 std dev definition was chosen instead (e.g. concurs with previous studies), and how far does it differ from the bottom/top 10th percentile definition, in practice?

*The reviewer is correct that using the 10^th^ and 90^th^ percentiles of observed birthweights is a commonly used definition of SGA and LGA, respectively. In previous sibling comparisons of perinatal health after assisted reproduction, observed birthweights were used to define SGA in the studies that included this outcome: In two studies, -2 standard deviations of the study populations’ own birthweight distributions were used(1, 2). Luke et al used national US standards of observed birthweight for gestational age, and defined SGA as below -1.28 standard deviation, corresponding to the 10^th^ percentile (3). Seggers et al. used Dutch reference curves of observed birthweight, defining weight below the 5^th^ percentile as SGA (4). The two remaining sibling studies used absolute cut-offs of low birthweight independent of gestational age (<2500 or <1500 grams) (5, 6).*

*The limitations of using observed birthweight have been addressed by Marsal et al. (1), who estimated expected birthweight according to gestational age from intrauterine (ultrasound) measurements of ongoing, presumably healthy pregnancies. Preterm birth often results from pregnancy complications or fetal conditions that can also affect fetal growth (7). In consequence, the observed birthweights in these preterm deliveries are not representative of the expected fetal weight distribution for healthy pregnancies at a given gestational age. In the study by Marsal et al. showed that the majority of preterm born infants’ observed birthweights fell below the estimated mean intrauterine weight for gestational age, resulting in a lower proportion of preterm born infants with SGA when using observed birthweight compared to intrauterine growth curves. In our study, where a high proportion of our ART-conceived participants are born preterm, we believe that using intrauterine expected weight standards rather than observed birthweight will give the most valid estimates. Furthermore, the study by Marsal et al. was based on pregnancies among Danish and Swedish women during the first part of our study period and are currently used with a +/- 22% limit in clinical practice in Sweden (8). Despite a small number of participants, the estimated growth patterns are very similar to those of a larger, more recent Norwegian population(9).*

*The use of ±22% as the limits for LGA and SGA, corresponds approximately to the 2.3 and 97.7 percentiles. Using the 10^th^ and 90^th^ percentile from an intrauterine standard would increase sensitivity, but at the cost of specificity by including a large number of constitutionally small or large foetuses/neonates as SGA or LGA(10). However, the differences in mean z-score between fresh-ET, frozen-ET and natural conception (Table 2) support that our conclusions are not dependent on a specific cut-off for SGA and LGA.*

*To illustrate the differences between the observed and estimated birthweights, we have plotted the observed birthweight in Main Sample 1 along with the Marsal standard. The areas in the graphs below correspond to the range of birthweights considered as appropriate for gestational age (AGA).*

*We have not made any changes to our definition of SGA and LGA in the paper and hope the reviewer agrees with our rationale. However, we have clarified in the discussion (Comparison with other studies) our chosen definition of SGA/LGA compared to definitions in previous studies:*

*While we defined birthweight for gestational age based on intrauterine growth curves [26], previous studies used observed birthweight and different criteria for large and small for gestational age [18-22]. As preterm birth often results from pregnancy complications that can affect fetal growth [39], the observed birthweights in preterm deliveries are not representative of the normal fetal weight distribution for healthy pregnancies at a given gestational age.*

2. For Sensitivity Analysis 2 in Figure 1, n=1,972,012 for naturally conceived infants, but n=1,982,791 in Supplementary Tables 5 & 6. This might be addressed.

*Thank you for spotting this mistake. For sensitivity analysis concerning children born by natural conception and within 3-year interval, the correct number of naturally conceived infants is 1 982 791. This has now been corrected in Figure 1.*

Minor issues:

3. In the abstract, "95% 70 to 94" might be "95% CI".

*Thank you for noticing, we have inserted “CI” where it was missing in the abstract.*

4. The abbreviations SGA, LGA, PTB and VPTB (probably relating to gestational age and preterm births) in Figure 2, might be defined at some prior point in the paper (possibly within the figure caption definitions, as done for natural conception for example)

*Thank you for noticing that this was missing. We have now removed the abbreviations in Figure 2 and write the outcomes using full names instead.*

*** Reviewer #2:

General comments

This is a retrospective cohort study within sub-ship analysis examining the perinatal outcome of babies born after fresh-ET and frozen-ET versus natural conception Due to the well-known quality of ART-registration in Denmark, Norway and Sweden the authors can provide very interesting information on perinatal outcoma after assisted reproduction.

The number of 33 056 offspring siblings with the same mother with at least two different conception methods is very important. Above this not only fresh-ET and frozen-ET offspring are compared, buth both groups are als compared with natural conception offspring in a huge number of patients.

I believe that this retrospective analysis, with even data of the paternal identity in 98 % of cases, deliveres results uncomparable with previous reports, not only due to the amount of cases, but also due to the methods used to prevent as much as possible confounding factors including socioeconomic status.

This paper can be regarded as a major contribution in our understanding how assisted reproduction can influence perinatal outcome results when compared to natural conception.

Specific comments

Only two comments:

Why did the authors looked at mean birthweight and small for getational age. In the literature <2.5kg and < 1.5 kg is mostly used.

*The main reason for choosing small and large for gestational age as outcomes rather than absolute weight limits such as <2500, <1500 or >4500 grams, was the higher risk of preterm birth in assisted reproduction. Given the strong association between gestational age and birthweight (also illustrated in the figures above), a higher risk of e.g. birthweight <2500 grams after assisted reproduction could result from both growth restriction and from preterm birth and separating the two would be challenging. Although the mentioned cut-offs have often been used in clinical practice, we believe that taking the available detailed information on gestational age into consideration gives a better opportunity to address growth more directly.*

*Using mean birthweight (in grams and z-score) and mean gestational age, we also compared the overall distributions of these factors without the need to specify a cut-off. This is particularly relevant in sibling studies because the fixed effects logistic model (within estimates for binary outcomes) uses only sibling groups that are discordant for the outcome.*

*Therefore, we have not made any changes to our outcome measures in the analysis or paper and hope the reviewer agree with our reasoning.*

There is no mention of the influence of different culture media on birthweight in the discussion. I assume that this parameter could not be examined.

*Unfortunately, we had no information on which culture media were used. We have added this as a limitation of our study (Discussion, strengths and limitation, end of 2^nd^ paragraph).* *It should be noted that culture media differ between clinics and over time, but not specifically between fresh-ET and frozen-ET, which makes it less likely that our results could be attributed to culture media.*

*** Reviewer #3:

This is the largest study investigating perinatal outcomes following fresh and frozen embryo transfers (ETs) compared with natural conception (NC) within siblings. The sample size is extremely larger (33,056 sibling pairs) than other sibling analysis studies. Further, the study stratified the analysis according to fresh/frozen status, which have not been done in most of previous studies. Though significant associations between ART and perinatal outcomes were attenuated and did not become significant within sib-ship analysis in many previous sibling studies, , this study found that significant association between fresh ET and LBW, SGA, and between frozen ET and higher birthweight and LGA. which was similar in population analysis. Only inconsistent result between population analysis and sibling analysis was very preterm birth.

The author took a strict strategy for sample selection to manage selection bias and confounding. Further, they repeated several sensitivity analyses to test the robustness of the associations. I have only several minor points that should be addressed before publication.

1. Discussion (P 18): Though the author mentioned about other studies for comparison, I do not think those studies are broadly consistent results. One recent study from the Lancet (Goisis et al.) demonstrated significant associations were very highly attenuated after statistical adjustment. Another study from the Netherlands (Seggers et al.) using 1813  sibling pairs demonstrated null association. Although the author mentioned the direction of the association was similar, non-significant associations did not have any directions. Further, conclusions of those previous studies were opposite to the current study. Thus, I recommend to describe more in detail about the discrepancy for study results, potential reasons and future implication on it.

*We have now elaborated further on differences between previous studies in the Discussion (Comparisons with other studies, p19)). To help the reader compare the results across studies, we have summarized some of them in a Supplementary table (Supplementary Table 13). We have included results on mean difference in birthweight and mean difference in gestational age, which are the two comparisons presented in all studies. For dichotomous outcomes, there are differences in definition between the studies as mentioned above, making a direct comparison more complicated. For some of the studies, we have changed the reference group (i.e. inverted the estimate) to ensure that all comparisons have the same reference group (natural conception in comparison of ART vs natural conception and Fresh-ET in comparisons of Frozen-ET vs Fresh-ET). Even though most individual studies did not show statistically significant adjusted estimates, we believe that they are compatible and collectively point in the direction of lower birthweight and shorter gestation in ART overall. These studies are performed in populations dominated by Fresh-ET and may therefore be considered as consistent with the results from our study for Fresh-ET vs natural conception. None of the studies compared Frozen-ET to natural conception, but the two previous studies that compared Frozen-ET to Fresh-ET show larger birthweights in Frozen-ET regardless of birth order, whereas the results for gestational age are less clear. This is also consistent with our results.*

**References**

1.	Romundstad LB, Romundstad PR, Sunde A, von Düring V, Skjaerven R, Gunnell D, et al. Effects of technology or maternal factors on perinatal outcome after assisted fertilisation: a population-based cohort study. Lancet. 2008;372(9640):737-43.

2.	Dhalwani NN, Boulet SL, Kissin DM, Zhang Y, McKane P, Bailey MA, et al. Assisted reproductive technology and perinatal outcomes: conventional versus discordant-sibling design. Fertil Steril. 2016;106(3):710-6.e2.

3.	Luke B, Brown MB, Wantman E, Stern JE, Toner JP, Coddington CC, 3rd. Increased risk of large-for-gestational age birthweight in singleton siblings conceived with in vitro fertilization in frozen versus fresh cycles. J Assist Reprod Genet. 2017;34(2):191-200.

4.	Seggers J, Pontesilli M, Ravelli ACJ, Painter RC, Hadders-Algra M, Heineman MJ, et al. Effects of in vitro fertilization and maternal characteristics on perinatal outcomes: a population-based study using siblings. Fertil Steril. 2016;105(3):590-8.e2.

5.	Henningsen AK, Pinborg A, Lidegaard Ø, Vestergaard C, Forman JL, Andersen AN. Perinatal outcome of singleton siblings born after assisted reproductive technology and spontaneous conception: Danish national sibling-cohort study. Fertil Steril. 2011;95(3):959-63.

6.	Goisis A, Remes H, Martikainen P, Klemetti R, Myrskylä M. Medically assisted reproduction and birth outcomes: a within-family analysis using Finnish population registers. Lancet. 2019;393(10177):1225-32.

7.	Goldenberg RL, Culhane JF, Iams JD, Romero R. Epidemiology and causes of preterm birth. Lancet. 2008;371(9606):75-84.

8.	Lindström L, Ageheim M, Axelsson O, Hussain-Alkhateeb L, Skalkidou A, Bergman E. Swedish intrauterine growth reference ranges of biometric measurements of fetal head, abdomen and femur. Sci Rep. 2020;10(1):22441.

9.	Johnsen SL, Rasmussen S, Wilsgaard T, Sollien R, Kiserud T. Longitudinal reference ranges for estimated fetal weight. Acta Obstet Gynecol Scand. 2006;85(3):286-97.

10.	Alberry M, Soothill P. Management of fetal growth restriction. Arch Dis Child Fetal Neonatal Ed. 2007;92(1):F62-7.
